# Rough Atanassov's Intuitionistic Fuzzy Sets Model over Two Universes and Its Applications

**DOI:** 10.1155/2014/348683

**Published:** 2014-05-13

**Authors:** Shuqun Luo, Weihua Xu

**Affiliations:** School of Mathematics and Statistics, Chongqing University of Technology, Chongqing 400054, China

## Abstract

Recently, much attention has been given to the rough set models based on two universes. And many rough set models based on two universes have been developed from different points of view. In this paper, a novel model, that is, rough Atanassov's intuitionistic fuzzy sets model over two different universes, is firstly proposed from Atanassov's intuitionistic point of view. We study some important properties of approximation operators and investigate the rough degree in the novel model. Furthermore, an illustrated example is employed to demonstrate the conceptual arguments of the model. Finally, rough Atanassov's intuitionistic fuzzy sets approach to decision is presented in the generalized approximation space over two universes by considering the problem about how to arrange patients to see the doctor reasonably, from which it can be found that the method is valuable and useful in real life.

## 1. Introduction

Rough set theory, originally proposed by Pawlak in the early 1980s [1–3] as a useful tool for treating with uncertainty or imprecision information, has been successfully applied in the fields of artificial intelligence, pattern recognition, medical diagnosis, data mining, conflict analysis, algebra [4–12], and so on. In recent years, the rough set theory has aroused a great deal of interest among more and more researchers.

It is widely accepted that the theory of rough sets, which is very important to construct a pair of upper and lower approximation operators, is based on available information. In the Pawlak approximation space, the lower and upper approximations of arbitrary subset of the universe of discourse can be represented directly. The lower approximation is the union of all equivalence classes, which are classes induced by the equivalence relation on the universe and included in the given set. The upper approximation is the union of all equivalence classes, which are classes induced by the equivalence relation on the universe having a nonempty intersection with the given set. So, the equivalence relation is a key and primitive notion in Pawlak's rough set model.

In the Pawlak approximation space, the equivalence relation is a very restrictive condition and the sets used are classical sets, so the application domain of rough set model is narrowed. In 1986, Atanassov introduced the concept of Atanassov's intuitionistic fuzzy sets. Atanassov's intuitionistic fuzzy set was considered as a generalization of fuzzy set and had been found to be very useful to deal with vagueness. Atanassov thought that Atanassov's intuitionistic fuzzy set was characterized by a pair of functions, the membership function and the nonmembership function valued in [0,1]. The degrees of membership and nonmembership are independent. So, Atanassov's intuitionistic fuzzy set is more suitable and precise to represent the essence of vagueness and is more useful than fuzzy set in dealing with imperfect information. Nowadays, many excellent works over single universe have been achieved. For example, Zhou and Wu [13] presented the rough approximations of Atanassov's intuitionistic fuzzy sets in crisp and fuzzy approximation spaces over single universe in which both constructive and axiomatic approaches are used. Zhang [14] generalized an interval-valued rough Atanassov's intuitionistic fuzzy (IF) sets model by means of integrating the classical Pawlak rough set theory with interval-valued Atanassov's intuitionistic fuzzy set theory. More excellent results can be found in [15, 16].

Moreover, the study on the rough set model over two universes was done, and it has become one of the hottest researches in recent years for authors. Shen and Wang [17] researched the variable precision rough set model over two universes and investigated the properties. Yan et al. [18] established the model of rough set over dual universes. Sun et al. [19] proposed Atanassov's intuitionistic fuzzy rough set model over two universes with a constructive approach and discussed the basic properties of this model in fuzzy approximation space. More details about recent advancements of rough set model over two universes can be found in the literature [20–25]. In this paper, we will discuss rough Atanassov's intuitionistic fuzzy sets model over two universes in the generalized approximation space, investigate its measures, and study how to use it for serving our life.

The rest of the paper is organized as follows. The next section reviews the basic concepts of Atanassov's intuitionistic fuzzy sets, rough sets, and rough fuzzy sets. In the next section, rough Atanassov's intuitionistic fuzzy sets model is constructed in generalized approximation space over two universes. Moreover, rough Atanassov's intuitionistic fuzzy sets' cut sets and some important properties are presented. In Section 4, we mainly studied how to measure the uncertainty of rough Atanassov's intuitionistic fuzzy set over two universes. What is more, a general approach to decision making is established based on rough Atanassov's intuitionistic fuzzy sets over two universes with a problem of how to order the patients to see the doctor reasonably as the background for the application in Section 5. Finally, we draw brief conclusions and set further research directions in Section 6.

## 2. Preliminaries

In this section, we will review some necessary definitions and concepts required in the sequel of this paper.

### 2.1. Atanassov's Intuitionistic Fuzzy Sets

**Definition 1 (see [26, 27])**

Let *U* be a nonempty finite set. Atanassov's intuitionistic fuzzy set $\tilde{A}$ on *U* is an object having the form

(1) $$\begin{array}{c} \tilde{A}=\left\{\langle x,\mu_{\tilde{A}}\left(x\right),\nu_{\tilde{A}}\left(x\right)\rangle \;|\;x\in U\right\}, \end{array}$$

where $\mu_{\tilde{A}}:U\to [0,1]$ and $\nu_{\tilde{A}}:U\to [0,1]$ satisfy $0\leq \mu_{\tilde{A}}(x)+\nu_{\tilde{A}}(x)\leq 1$ for all *x* ∈ *U*.

$\mu_{\tilde{A}}(x)$ and $\nu_{\tilde{A}}(x)$ are, respectively, called the degrees of membership and nonmembership of the element *x* ∈ *U* to $\tilde{A}$.

The complement of Atanassov's intuitionistic fuzzy set $\tilde{A}$ is denoted by $∼\tilde{A}=\{\langle x,\nu_{\tilde{A}}(x),\mu_{\tilde{A}}(x)\rangle |x\in U\}$.

Let *IF*(*U*) denote the family of all Atanassov's intuitionistic fuzzy sets on *U*.

For any $\tilde{A},\tilde{B}\in IF(U)$, some basic operations on *IF*(*U*) are defined as follows:

1. $\tilde{A}\subseteq \tilde{B}\Leftrightarrow \mu_{\tilde{A}}(x)\leq \mu_{\tilde{B}}(x)$, $\nu_{\tilde{A}}(x)\geq \nu_{\tilde{B}}(x)$ for all *x* ∈ *U*,
2. $\tilde{A}=\tilde{B}\Leftrightarrow \mu_{\tilde{A}}(x)=\mu_{\tilde{B}}(x)$, $\nu_{\tilde{A}}(x)=\nu_{\tilde{B}}(x)$ for all *x* ∈ *U*,
3. $\tilde{A}\cap \tilde{B}=\{\langle x,\mu_{\tilde{A}}(x)\wedge \mu_{\tilde{B}}(x),\nu_{\tilde{A}}(x)\vee \nu_{\tilde{B}}(x)\rangle |x\in U\}$,
4. $\tilde{A}\cup \tilde{B}=\{\langle x,\mu_{\tilde{A}}(x)\vee \mu_{\tilde{B}}(x),\nu_{\tilde{A}}(x)\wedge \nu_{\tilde{B}}(x)\rangle |x\in U\}$,
5. if *λ* > 0, then $\lambda \tilde{A}=\{\langle x,1-{\left(1-\mu_{\tilde{A}}(x)\right)}^{\lambda },{\left(\nu_{\tilde{A}}(x)\right)}^{\lambda }\rangle |x\in U\}$.

**Definition 2**

Let $\tilde{A}$ be Atanassov's intuitionistic fuzzy set over *U* and (*α*, *β*) ∈ *L*, *L* = {(*α*, *β*) | *α* ∈ [0,1], *β* ∈ [0,1], *α* + *β* ≤ 1}, and the (*α*, *β*)-level cut set of $\tilde{A}$, denoted by ${\tilde{A}}_{\alpha }^{\beta }$, is defined as follows:

(2) $$\begin{array}{c} {\tilde{A}}_{\alpha }^{\beta }=\left\{x\in U\;|\;\mu_{\tilde{A}}\left(x\right)\geq \alpha ,\nu_{\tilde{A}}\left(x\right)\leq \beta \right\}. \end{array}$$

${\tilde{A}}_{\alpha }=\{x\in U|\mu_{\tilde{A}}(x)\geq \alpha \}$ and ${\tilde{A}}_{\alpha +}=\{x\in U|\mu_{\tilde{A}}(x)>\alpha \}$ are, respectively, called the *α*-level cut set and the strong *α*-level cut set of membership generated by $\tilde{A}$. And ${\tilde{A}}^{\beta }=\{x\in U|\nu_{\tilde{A}}(x)\leq \beta \}$ and ${\tilde{A}}^{\beta +}=\{x\in U|\nu_{\tilde{A}}(x)<\beta \}$ are, respectively, referred to as the *β*-level cut set and the strong *β*-level cut set of nonmembership generated by $\tilde{A}$.

What is more, other types of cut sets and strong cut sets of Atanassov's intuitionistic fuzzy set $\tilde{A}$ are denoted, for example, ${\tilde{A}}_{\alpha +}^{\beta +}=\{x\in U|\mu_{\tilde{A}}(x)>\alpha ,\nu_{\tilde{A}}(x)<\beta \}$, which is called the (*α* + , *β*+)-level cut set of $\tilde{A}$.

### 2.2. Fuzzy Rough Sets Model

**Definition 3 (see [28])**

Let $\left(U,\tilde{R}\right)$ be a fuzzy approximation space and $\tilde{A}$ be a fuzzy subset of *U*. The lower approximation and upper approximation are denoted by $\underline{\tilde{R}}(\tilde{A})$ and $\bar{\tilde{R}}(\tilde{A})$, respectively. The memberships of *x* to $\tilde{A}$ are defined as

(3) $$\begin{array}{c} \underline{\tilde{R}}\left(\tilde{A}\right)\left(x\right)=\wedge \left\{\tilde{A}\left(y\right)\vee \left(1-\tilde{R}\left(x,y\right)\right)\;|\;y\in U\right\},\;x\in U,\\ \bar{\tilde{R}}\left(\tilde{A}\right)\left(x\right)=\vee \left\{\tilde{A}\left(y\right)\wedge \left(1-\tilde{R}\left(x,y\right)\right)\;|\;y\in U\right\},\;x\in U, \end{array}$$

where “∨” and “∧” mean “min” and “max” operators, respectively, and $\tilde{A}(y)$ is the membership of *y* with respect to $\tilde{A}$. The pair $\left(\underline{\tilde{R}}(\tilde{A}),\bar{\tilde{R}}(\tilde{A})\right)$ is called a fuzzy rough set.

### 2.3. Rough Atanassov's Intuitionistic Fuzzy Sets Model over One Universe

**Definition 4 (see [13])**

Let (*U*, *R*) be a generalized approximation space, for any $\tilde{A}=\{\langle x,\mu_{\tilde{A}}(x),\nu_{\tilde{A}}(x)\rangle |x\in U\}\in IF(U)$. The upper and lower approximations of $\tilde{A}$, denoted by $\bar{R}(\tilde{A})$ and $\underline{R}(\tilde{A})$, are, respectively, defined as follows:

(4) $$\begin{array}{c} \bar{R}\left(\tilde{A}\right)=\left\{\langle x,\mu_{\bar{R}(\tilde{A})}\left(x\right),\nu_{\bar{R}(\tilde{A})}\left(x\right)\rangle \;|\;x\in U\right\},\\ \underline{R}\left(\tilde{A}\right)=\left\{\langle x,\mu_{\underline{R}(\tilde{A})}\left(x\right),\nu_{\underline{R}(\tilde{A})}\left(x\right)\rangle \;|\;x\in U\right\}, \end{array}$$

where

(5) $$\begin{array}{c} \mu_{\bar{R}(\tilde{A})}\left(x\right)=\overset{}{\underset{y\in R_{s}(x)}{⋁}}\mu_{\tilde{A}}\left(y\right),\;\;\nu_{\bar{R}(\tilde{A})}\left(x\right)=\overset{}{\underset{y\in R_{s}(x)}{⋀}}\nu_{\tilde{A}}\left(y\right),\\ \mu_{\underline{R}(\tilde{A})}\left(x\right)=\overset{}{\underset{y\in R_{s}\left(x\right)}{⋀}}\mu_{\tilde{A}}\left(y\right),\;\;\nu_{\underline{R}(\tilde{A})}\left(x\right)=\overset{}{\underset{y\in R_{s}\left(x\right)}{⋁}}\nu_{\tilde{A}}\left(y\right), \end{array}$$

where “∨” and “∧” mean “min” and “max” operators, respectively, and $\mu_{\tilde{A}}(y)$, $\nu_{\tilde{A}}(y)$ are the membership and nonmembership of *y* with respect to $\tilde{A}$. The pair $\left(\underline{R}(\tilde{A}),\bar{R}(\tilde{A})\right)$ is called a fuzzy rough set in a generalized approximation space.

### 2.4. Rough Fuzzy Sets Model over Two Universes

**Definition 5 (see [29])**

Let (*U*, *V*, *R*) be a generalized approximation space over two universes, and for any $\tilde{A}\in F(U)$, $\tilde{B}\in F(V)$, denote

(6) R_U(A~)(y)=min⁡⁡{A~(x) ∣ x∈Rp(y)},R¯U(A~)(y)=max⁡⁡{A~(x) ∣ x∈Rp(y)},y∈V;R_V(B~)(x)=min⁡⁡{B~(y) ∣ y∈Rs(x)},R¯V(B~)(x)=max⁡⁡{B~(y) ∣ y∈Rs(x)},x∈U.

Then, ${\underline{R}}_{U}(\tilde{A})$ and ${\bar{R}}_{U}(\tilde{A})$ are called the lower and upper approximations of fuzzy set $\tilde{A}$ in *F*(*U*), and ${\underline{R}}_{V}(\tilde{B})$ and ${\bar{R}}_{V}(\tilde{B})$ are called the lower and upper approximations of fuzzy set $\tilde{B}$ in *F*(*V*), respectively. (${\underline{R}}_{U}(\tilde{A}),{\bar{R}}_{U}(\tilde{A})$) and (${\underline{R}}_{V}(\tilde{B}),{\bar{R}}_{V}(\tilde{B})$) are called rough fuzzy sets in generalized approximation space over two universes.

## 3. Rough Atanassov's Intuitionistic Fuzzy Sets Model over Two Universes

In this section, we will introduce rough Atanassov's intuitionistic fuzzy sets model over the different universes and discuss some important properties of rough Atanassov's intuitionistic fuzzy sets.

### 3.1. Construction of Rough Atanassov's Intuitionistic Fuzzy Sets

Let *U*, *V* be nonempty finite universes, and a subset *R* ∈ *P*(*U* × *V*) (i.e., *R*: *U* × *V* → {0,1}) is called a binary relation from *U* to *V*.

In general, if *U* = *V*, *R* is called the binary relation over single universe. If *R* satisfies reflexivity, symmetry, and transitivity, then we say *R* is an equivalence relation. But in generalized approximation space over two universes, *R* is a binary relation from *U* to *V* and then *R* must not be equivalence relation.

**Definition 6 (see [22])**

Let *R* be a crisp binary relation on the universes *U* and *V*. Then,

1. *R* is serial if, for any *x* ∈ *U*, there exists *y* ∈ *V*, s.t. *R*(*x*, *y*) = 1,
2. *R* is reverse serial if, for any *y* ∈ *V*, there exists *x* ∈ *U*, s.t. *R*(*x*, *y*) = 1.

**Definition 7**

Let (*U*, *V*, *R*) be a generalized approximation space over two universes, and for any $\tilde{A}\in IF(U)$, $\tilde{B}\in IF(V)$, denote

(7) $$\begin{array}{c} {\underline{R}}_{U}\left(\tilde{A}\right)=\left\{\langle y,\mu_{{\underline{R}}_{U}(\tilde{A})}\left(y\right),\nu_{{\underline{R}}_{U}(\tilde{A})}\left(y\right)\rangle \;|\;y\in V\right\},\\ {\bar{R}}_{U}\left(\tilde{A}\right)=\left\{\langle y,\mu_{{\bar{R}}_{U}(\tilde{A})}\left(y\right),\nu_{{\bar{R}}_{U}(\tilde{A})}\left(y\right)\rangle \;|\;y\in V\right\},\\ {\underline{R}}_{V}\left(\tilde{B}\right)=\left\{\langle x,\mu_{{\underline{R}}_{V}(\tilde{B})}\left(x\right),\nu_{{\underline{R}}_{V}(\tilde{B})}\left(x\right)\rangle \;|\;x\in U\right\},\\ {\bar{R}}_{V}\left(\tilde{B}\right)=\left\{\langle x,\mu_{{\bar{R}}_{V}(\tilde{B})}\left(x\right),\nu_{{\bar{R}}_{V}(\tilde{B})}\left(x\right)\rangle \;|\;x\in U\right\}, \end{array}$$

where

(8) $$\begin{array}{c} \mu_{{\underline{R}}_{U}(\tilde{A})}\left(y\right)=\overset{}{\underset{x\in R_{p}(y)}{⋀}}\mu_{\tilde{A}}\left(x\right),\;\;\nu_{{\underline{R}}_{U}(\tilde{A})}\left(y\right)=\overset{}{\underset{x\in R_{p}(y)}{⋁}}\nu_{\tilde{A}}\left(x\right),\\ \mu_{{\bar{R}}_{U}(\tilde{A})}\left(y\right)=\overset{}{\underset{x\in R_{p}(y)}{⋁}}\mu_{\tilde{A}}\left(x\right),\;\;\nu_{{\bar{R}}_{U}(\tilde{A})}\left(y\right)=\overset{}{\underset{x\in R_{p}(y)}{⋀}}\nu_{\tilde{A}}\left(x\right),\\ \mu_{{\underline{R}}_{V}(\tilde{B})}\left(x\right)=\overset{}{\underset{y\in R_{s}(x)}{⋀}}\mu_{\tilde{B}}\left(y\right),\;\;\nu_{{\underline{R}}_{V}(\tilde{B})}\left(x\right)=\overset{}{\underset{y\in R_{s}(x)}{⋁}}\nu_{\tilde{B}}\left(y\right),\\ \mu_{{\bar{R}}_{V}(\tilde{B})}\left(x\right)=\overset{}{\underset{y\in R_{s}\left(x\right)}{⋁}}\mu_{\tilde{B}}\left(y\right),\;\;\nu_{{\bar{R}}_{V}(\tilde{B})}\left(x\right)=\overset{}{\underset{y\in R_{s}\left(x\right)}{⋀}}\nu_{\tilde{B}}\left(y\right). \end{array}$$

Then, ${\underline{R}}_{U}(\tilde{A})$ and ${\bar{R}}_{U}(\tilde{A})$ are called the lower and upper approximations of Atanassov's intuitionistic fuzzy set $\tilde{A}$ in *IF*(*U*), and ${\underline{R}}_{V}(\tilde{B})$ and ${\bar{R}}_{V}(\tilde{B})$ are called the lower and upper approximations of Atanassov's intuitionistic fuzzy set $\tilde{B}$ in *IF*(*V*). $\left({\underline{R}}_{U}(\tilde{A}),{\bar{R}}_{U}(\tilde{A})\right)$ and $\left({\underline{R}}_{V}(\tilde{B}),{\bar{R}}_{V}(\tilde{B})\right)$ are called rough Atanassov's intuitionistic fuzzy sets over the universes *U* and *V*.

Furthermore, we also define the positive region $\text{po}{\text{s}}_{R_{U}}(\tilde{A})$, $\text{po}{\text{s}}_{R_{V}}(\tilde{B})$, negative region $\text{ne}{\text{g}}_{R_{U}}(\tilde{A})$, $\text{ne}{\text{g}}_{R_{V}}(\tilde{B})$, and boundary region $\text{b}{\text{n}}_{R_{U}}(\tilde{A})$, $\text{b}{\text{n}}_{R_{V}}(\tilde{B})$ of $\tilde{A},\tilde{B}$ about *R*_*U*_, *R*_*V*_ on the universe *U*, *V* as follows, respectively:

(9) $$\begin{array}{c} \text{po}{\text{s}}_{R_{U}}\left(\tilde{A}\right)={\underline{R}}_{U}\left(\tilde{A}\right),\;\;\text{ne}{\text{g}}_{R_{U}}\left(\tilde{A}\right)=∼{\bar{R}}_{U}\left(\tilde{A}\right),\\ \text{b}{\text{n}}_{R_{U}}\left(\tilde{A}\right)={\bar{R}}_{U}\left(\tilde{A}\right)\cap ∼{\underline{R}}_{U}\left(\tilde{A}\right);\\ \text{po}{\text{s}}_{R_{V}}\left(\tilde{B}\right)={\underline{R}}_{V}\left(\tilde{B}\right),\;\;\text{ne}{\text{g}}_{R_{V}}\left(\tilde{B}\right)=∼{\bar{R}}_{V}\left(\tilde{B}\right),\\ \text{b}{\text{n}}_{R_{V}}\left(\tilde{B}\right)={\bar{R}}_{V}\left(\tilde{B}\right)\cap ∼{\underline{R}}_{V}\left(\tilde{B}\right). \end{array}$$

If for any *y* ∈ *V* (resp., *x* ∈ *U*), ${\underline{R}}_{U}(\tilde{A})={\bar{R}}_{U}(\tilde{A})$ (resp., ${\underline{R}}_{V}(\tilde{B})={\bar{R}}_{V}(\tilde{B})$), then Atanassov's intuitionistic fuzzy set $\tilde{A}$ (resp., $\tilde{B}$) is Atanassov's intuitionistic fuzzy definable set about the generalized approximation space (*U*, *V*, *R*). Otherwise, Atanassov's intuitionistic fuzzy set $\tilde{A}$ (resp., $\tilde{B}$) is a rough set about the generalized approximation space (*U*, *V*, *R*) over two universes.

**Example 8**

Let (*U*, *V*, *R*) be a generalized approximation space over two universes. Let *U*, *V* be two nonempty finite universes, and let *U* denote the symptom set, and let *V* denote the disease set. Suppose *U* = {*x*_1_, *x*_2_, *x*_3_, *x*_4_, *x*_5_}, *V* = {*y*_1_, *y*_2_, *y*_3_, *y*_4_, *y*_5_}, where each *x*_*i*_  (*i* = 1,2,…, 5) stands for one symptom, but each *y*_*i*_  (*i* = 1,2,…, 5) stands for a disease. Assume *R* be a binary relation on *U* × *V*, for any *x*_*i*_ ∈ *U*, if there exists *y* ∈ *V*, so the relation can be understood that if a person has a symptom *x*_*i*_, so he had possibly suffered from a disease *y*_*i*_.

Now, we can define the relation *R* as follows:

(10) R={(x1,y1),(x1,y2),(x1,y3),(x2,y3),(x3,y4),(x4,y1),(x4,y2),(x5,y5)}.

From *R*, we can see that *R* is serial and reverse serial, and we can obtain

(11) $$\begin{array}{c} R_{P}\left(y_{1}\right)=\left\{x_{1},x_{4}\right\},\;\;R_{P}\left(y_{2}\right)=\left\{x_{1},x_{4}\right\},\\ R_{P}\left(y_{3}\right)=\left\{x_{1},x_{2}\right\},\;\;R_{P}\left(y_{4}\right)=\left\{x_{3}\right\},\\ R_{P}\left(y_{5}\right)=\left\{x_{5}\right\}. \end{array}$$

Suppose a person $\tilde{A}$ has the symptom conditions, and we can describe by Atanassov's intuitionistic fuzzy set

(12) A~={〈x1,0.8,0.1〉,〈x2,0.6,0.1〉,〈x3,0.2,0.8〉,〈x4,0.6,0.1〉,〈x5,0.1,0.6〉}.

By Definition 7, we can obtain

(13) R_U(A~)={〈y1,0.6,0.1〉,〈y2,0.6,0.1〉,〈y3,0.6,0.1〉,〈y4,0.2,0.8〉,〈y5,0.1,0.6〉},R¯U(A~)={〈y1,0.8,0.1〉,〈y2,0.8,0.1〉,〈y3,0.8,0.1〉,〈y4,0.2,0.8〉,〈y5,0.1,0.6〉}.

From the lower and upper approximations of $\tilde{A}$, we can draw a conclusion that the person must have had the diseases *y*_1_, *y*_2_, *y*_3_, *y*_4_, *y*_5_ at the degrees (0.6,0.1), (0.6,0.1), (0.6,0.1), (0.2,0.8), and (0.1,0.6), respectively. And the person may be have had the diseases *y*_1_, *y*_2_, *y*_3_, *y*_4_, *y*_5_ at the degrees (0.8,0.1), (0.8,0.1), (0.8,0.1), (0.2,0.8), and (0.1,0.6), respectively.

**Remark 9**

In a generalized approximation space over two universes, we can find out that the lower and upper approximations of Atanassov's intuitionistic fuzzy set $\tilde{A}\in IF(U)$ belong to *IF*(*V*), and the lower and upper approximations of Atanassov's intuitionistic fuzzy set $\tilde{B}\in IF(V)$ belong to *IF*(*U*). This property is different from the lower and upper approximations over single universe. What is more, we can obtain other properties in the following.

### 3.2. Corresponding Properties of Rough Atanassov's Intuitionistic Fuzzy Sets

**Theorem 10**

Let (*U*, *V*, *R*) be a generalized approximation space over two universes, and for any $\tilde{A},{\tilde{A}}^{\prime }\in IF(U)$, $\tilde{B},{\tilde{B}}^{\prime }\in IF(V)$, one has the following properties:

(1) ${\underline{R}}_{U}(\tilde{A})=∼{\bar{R}}_{U}(∼\tilde{A})$, ${\bar{R}}_{U}(\tilde{A})=∼{\underline{R}}_{U}(∼\tilde{A})$;
 
${\underline{R}}_{V}(\tilde{B})=∼{\bar{R}}_{V}(∼\tilde{B})$, ${\bar{R}}_{V}(\tilde{B})=∼{\underline{R}}_{V}(∼\tilde{B})$;
(2) ${\underline{R}}_{U}(\tilde{A}\cap {\tilde{A}}^{\prime })={\underline{R}}_{U}(\tilde{A})\cap {\underline{R}}_{U}({\tilde{A}}^{\prime })$, ${\bar{R}}_{U}(\tilde{A}\cup {\tilde{A}}^{\prime })={\bar{R}}_{U}(\tilde{A})\cup {\bar{R}}_{U}({\tilde{A}}^{\prime })$;
 
${\underline{R}}_{V}(\tilde{B}\cap {\tilde{B}}^{\prime })={\underline{R}}_{V}(\tilde{B})\cap {\underline{R}}_{V}({\tilde{B}}^{\prime })$, ${\bar{R}}_{V}(\tilde{B}\cup {\tilde{B}}^{\prime })={\bar{R}}_{V}(\tilde{B})\cup {\bar{R}}_{V}({\tilde{B}}^{\prime })$;
(3) ${\tilde{A}}^{\prime }\subseteq \tilde{A}\Rightarrow {\underline{R}}_{U}({\tilde{A}}^{\prime })\subseteq {\underline{R}}_{U}(\tilde{A}),{\bar{R}}_{U}({\tilde{A}}^{\prime })\subseteq {\bar{R}}_{U}(\tilde{A})$;
 
${\tilde{B}}^{\prime }\subseteq \tilde{B}\Rightarrow {\underline{R}}_{V}({\tilde{B}}^{\prime })\subseteq {\underline{R}}_{V}(\tilde{B}),{\bar{R}}_{V}({\tilde{B}}^{\prime })\subseteq {\bar{R}}_{V}(\tilde{B})$;
(4) ${\underline{R}}_{U}(\tilde{A}\cup {\tilde{A}}^{\prime })⊇{\underline{R}}_{U}(\tilde{A})\cup {\underline{R}}_{U}({\tilde{A}}^{\prime })$, ${\bar{R}}_{U}(\tilde{A}\cap {\tilde{A}}^{\prime })\subseteq {\bar{R}}_{U}(\tilde{A})\cap {\bar{R}}_{U}({\tilde{A}}^{\prime })$;
 
${\underline{R}}_{V}(\tilde{B}\cup {\tilde{B}}^{\prime })⊇{\underline{R}}_{V}(\tilde{B})\cup {\underline{R}}_{V}({\tilde{B}}^{\prime })$, ${\bar{R}}_{V}(\tilde{B}\cap {\tilde{B}}^{\prime })\subseteq {\bar{R}}_{V}(\tilde{B})\cap {\bar{R}}_{V}({\tilde{B}}^{\prime })$;
(5) ${\underline{R}}_{U}(\tilde{A})\subseteq {\bar{R}}_{U}(\tilde{A})$, ${\underline{R}}_{V}(\tilde{B})\subseteq {\bar{R}}_{V}(\tilde{B})$.

**Proof**

We only need to prove the first part of each property as the similarity of the above properties.

(1) According to Definition 7, we can obtain

(14) R_U(∼A~) ={〈y,⋀x∈Rp(y)μ∼A~(x),⋁x∈Rp(y)ν∼A~(x)〉 ∣ y∈V} ={〈y,⋀x∈Rp(y)νA~(x),⋁x∈Rp(y)μA~(x)〉 ∣ y∈V} =∼R¯U(A~).

So, we can have ${\bar{R}}_{U}(\tilde{A})=∼{\underline{R}}_{U}(∼\tilde{A})$.

The property ${\underline{R}}_{U}(\tilde{A})=∼{\bar{R}}_{U}(∼\tilde{A})$ can be proved similarly.

(2) We can have

(15) R_U(A~∩A~′)={〈y,⋀x∈Rp(y)μA~∩A~′(x),⋁x∈Rp(y)νA~∩A~′(x)〉 ∣ y∈V}={〈y,⋀x∈Rp(y)(μA~(x)∧μA~(x)),⋁x∈Rp(y)(μA~(x)∨μA~(x))〉 ∣ y∈V}={〈y,⋀x∈Rp(y)μA~(x)∧⋀x∈Rp(y)μA~′(x),⋁x∈Rp(y)νA~(x)∨⋁x∈Rp(y)νA~′(x)〉 ∣ y∈V}=R_U(A~)∩R_U(A~′).

Hence, we can obtain ${\underline{R}}_{U}(\tilde{A}\cap {\tilde{A}}^{\prime })={\underline{R}}_{U}(\tilde{A})\cap {\underline{R}}_{U}({\tilde{A}}^{\prime })$.

(3) According to the definitions of Atanassov's intuitionistic fuzzy lower and fuzzy upper approximations, (3) holds.

(4) It is easy to prove it by property (3).

(5) For any $y\in {\underline{R}}_{U}(\tilde{A})$, we can have

(16) $$\begin{array}{c} \mu_{{\underline{R}}_{U}(\tilde{A})}\left(y\right)=\overset{}{\underset{x\in R_{p}(y)}{⋀}}\mu_{\tilde{A}}\left(x\right)\leq \overset{}{\underset{x\in R_{p}(y)}{⋁}}\mu_{\tilde{A}}\left(x\right),\\ \nu_{{\underline{R}}_{U}(\tilde{A})}\left(y\right)=\overset{}{\underset{x\in R_{p}(y)}{⋁}}\nu_{\tilde{A}}\left(x\right)\geq \overset{}{\underset{x\in R_{p}(y)}{⋀}}\nu_{\tilde{A}}\left(x\right). \end{array}$$

Therefore, ${\underline{R}}_{U}(\tilde{A})\subseteq {\bar{R}}_{U}(\tilde{A})$.

**Definition 11**

Let (*U*, *V*, *R*) be a generalized approximation space over two universes, and for any $\tilde{A}\in IF(U)$, $\tilde{B}\in IF(V)$, denote

(17) $$\begin{array}{c} {\underline{R}}_{U}\left({\tilde{A}}_{\alpha }^{\beta }\right)=\left\{y\;|\;R_{p}\left(y\right)\subseteq {\tilde{A}}_{\alpha }^{\beta }\right\},\\ {\bar{R}}_{U}\left({\tilde{A}}_{\alpha }^{\beta }\right)=\left\{y\;|\;R_{p}\left(y\right)\cap {\tilde{A}}_{\alpha }^{\beta }\neq \emptyset \right\};\\ {\underline{R}}_{V}\left({\tilde{B}}_{\alpha }^{\beta }\right)=\left\{x\;|\;R_{s}\left(x\right)\subseteq {\tilde{B}}_{\alpha }^{\beta }\right\},\\ {\bar{R}}_{V}\left({\tilde{B}}_{\alpha }^{\beta }\right)=\left\{x\;|\;R_{s}\left(x\right)\cap {\tilde{B}}_{\alpha }^{\beta }\neq \emptyset \right\}, \end{array}$$

where *α*, *β* ∈ [0,1], ${\underline{R}}_{U}({\tilde{A}}_{\alpha }^{\beta })$, and ${\bar{R}}_{U}({\tilde{A}}_{\alpha }^{\beta })$ are called the lower and upper approximation of ${\tilde{A}}_{\alpha }^{\beta }$ on the universe *U* and ${\underline{R}}_{V}({\tilde{B}}_{\alpha }^{\beta })$ and ${\bar{R}}_{V}({\tilde{B}}_{\alpha }^{\beta })$ are called the lower and upper approximations of ${\tilde{B}}_{\alpha }^{\beta }$ on the universe *V*.

**Example 12 (continued from Example 8)**

Let *α* = 0.8 and let *β* = 0.1; then, we have ${\tilde{A}}_{0.8}^{0.1}=\{x_{1}\}$, so the lower and upper approximations of ${\tilde{A}}_{0.8}^{0.1}$ can be presented as follows:

(18) $$\begin{array}{c} {\underline{R}}_{U}\left({\tilde{A}}_{0.8}^{0.1}\right)=\emptyset ,\\ {\bar{R}}_{U}\left({\tilde{A}}_{0.8}^{0.1}\right)=\left\{y_{1},y_{2},y_{3}\right\}. \end{array}$$

**Remark 13**

In Definition 7, we give the concepts of the lower approximation and upper approximation of ${\tilde{A}}_{\alpha }^{\beta }$ about *R* from the universe *U* to *V*. Similarly, we can also define the lower approximation and upper approximation of sets ${\tilde{A}}_{\alpha }$, ${\tilde{A}}_{\alpha +}$, ${\tilde{A}}^{\beta }$, ${\tilde{A}}^{\beta +}$, ${\tilde{A}}_{\alpha +}^{\beta }$, ${\tilde{A}}_{\alpha }^{\beta +}$, and ${\tilde{A}}_{\alpha +}^{\beta +}$ about *R* from the universe *U* to *V*. For example, we define the lower approximation and upper approximation of ${\tilde{A}}^{\beta +}$, ${\tilde{B}}^{\beta +}$ about *R* from the universe *U* to *V* as follows:

(19) $$\begin{array}{c} {\underline{R}}_{U}\left({\tilde{A}}^{\beta +}\right)=\left\{y\;|\;R_{p}\left(y\right)\subseteq {\tilde{A}}^{\beta +}\right\},\\ {\bar{R}}_{U}\left({\tilde{A}}^{\beta +}\right)=\left\{y\;|\;R_{p}\left(y\right)\cap {\tilde{A}}^{\beta +}\neq \emptyset \right\};\\ {\underline{R}}_{V}\left({\tilde{B}}^{\beta +}\right)=\left\{x\;|\;R_{s}\left(x\right)\subseteq {\tilde{B}}^{\beta +}\right\},\\ {\bar{R}}_{V}\left({\tilde{B}}^{\beta +}\right)=\left\{x\;|\;R_{s}\left(x\right)\cap {\tilde{B}}^{\beta +}\neq \emptyset \right\}. \end{array}$$

In the following discussion, without loss of generality, we only investigate the properties of ${\underline{R}}_{U}({\tilde{A}}_{\alpha }^{\beta })$, ${\bar{R}}_{U}({\tilde{A}}_{\alpha }^{\beta })$ and ${\underline{R}}_{V}({\tilde{B}}_{\alpha }^{\beta })$, ${\bar{R}}_{V}({\tilde{B}}_{\alpha }^{\beta })$. The corresponding properties can be extended to the other lower approximations and upper approximations; here, we will not list them one by one.

**Theorem 14**

Let (*U*, *V*, *R*) be a generalized approximation space over two universes; if *α*_1_ > *α*_2_, *β*_1_ < *β*_2_, one can obtain

(20) $$\begin{array}{c} {\underline{R}}_{U}\left({\tilde{A}}_{\alpha_{1}}^{\beta_{1}}\right)\subseteq {\underline{R}}_{U}\left({\tilde{A}}_{\alpha_{2}}^{\beta_{2}}\right),\;\;{\bar{R}}_{U}\left({\tilde{A}}_{\alpha_{1}}^{\beta_{1}}\right)\subseteq {\bar{R}}_{U}\left({\tilde{A}}_{\alpha_{2}}^{\beta_{2}}\right);\\ {\underline{R}}_{V}\left({\tilde{B}}_{\alpha_{1}}^{\beta_{1}}\right)\subseteq {\underline{R}}_{V}\left({\tilde{B}}_{\alpha_{2}}^{\beta_{2}}\right),\;\;{\bar{R}}_{V}\left({\tilde{B}}_{\alpha_{1}}^{\beta_{1}}\right)\subseteq {\bar{R}}_{V}\left({\tilde{B}}_{\alpha_{2}}^{\beta_{2}}\right). \end{array}$$

**Proof**

Since *α*_1_ > *α*_2_, *β*_1_ < *β*_2_, so ${\tilde{A}}_{\alpha_{1}}^{\beta_{1}}\subseteq {\tilde{A}}_{\alpha_{2}}^{\beta_{2}}$. For any $y\in {\underline{R}}_{U}({\tilde{A}}_{\alpha_{1}}^{\beta_{1}})$, we can have $R_{p}(y)\subseteq {\tilde{A}}_{\alpha_{1}}^{\beta_{1}}$. Thus, $R_{p}(y)\subseteq {\tilde{A}}_{\alpha_{2}}^{\beta_{2}}\Leftrightarrow y\in {\underline{R}}_{U}({\tilde{A}}_{\alpha_{2}}^{\beta_{2}})$ that is, ${\underline{R}}_{U}({\tilde{A}}_{\alpha_{1}}^{\beta_{1}})\subseteq {\underline{R}}_{U}({\tilde{A}}_{\alpha_{2}}^{\beta_{2}})$.

The properties ${\bar{R}}_{U}({\tilde{A}}_{\alpha_{1}}^{\beta_{1}})\subseteq {\bar{R}}_{U}({\tilde{A}}_{\alpha_{2}}^{\beta_{2}})$, ${\underline{R}}_{V}({\tilde{B}}_{\alpha_{1}}^{\beta_{1}})\subseteq {\underline{R}}_{V}({\tilde{B}}_{\alpha_{2}}^{\beta_{2}})$, and ${\bar{R}}_{V}({\tilde{B}}_{\alpha_{1}}^{\beta_{1}})\subseteq {\bar{R}}_{V}({\tilde{B}}_{\alpha_{2}}^{\beta_{2}})$ can be proved similarly.

**Example 15 (continued from Example 12)**

Let *α*_1_ = 0.8, *α*_2_ = 0.6, *β*_1_ = 0.1, and *β*_2_ = 0.2; then, we have ${\tilde{A}}_{0.6}^{0.2}=\{x_{1},x_{2},x_{4}\}$, so the lower and upper approximations of ${\tilde{A}}_{0.6}^{0.2}$ can be obtained as follows:

(21) $$\begin{array}{c} {\underline{R}}_{U}\left({\tilde{A}}_{0.6}^{0.2}\right)=\left\{y_{1},y_{2},y_{3}\right\},\\ {\bar{R}}_{U}\left({\tilde{A}}_{0.6}^{0.2}\right)=\left\{y_{1},y_{2},y_{3}\right\}. \end{array}$$

Then, ${\underline{R}}_{U}({\tilde{A}}_{0.8}^{0.1})\subseteq {\underline{R}}_{U}({\tilde{A}}_{0.6}^{0.2})$ and ${\bar{R}}_{U}({\tilde{A}}_{0.8}^{0.1})\subseteq {\bar{R}}_{U}({\tilde{A}}_{0.6}^{0.2})$ hold.

According to Definition 7, we can define two pairs of Atanassov's intuitionistic fuzzy sets as follows:

(22) $$\begin{array}{c} {\underline{R}}_{U}^{\prime }\left(\tilde{A}\right)=\left\{\langle y,\mu_{{\underline{R}}_{U}^{\prime }(\tilde{A})}\left(y\right),\nu_{{\underline{R}}_{U}^{\prime }(\tilde{A})}\left(y\right)\rangle \;|\;y\in V\right\},\\ {\bar{R}}_{U}^{\prime }\left(\tilde{A}\right)=\left\{\langle y,\mu_{{\bar{R}}_{U}^{\prime }(\tilde{A})}\left(y\right),\nu_{{\bar{R}}_{U}^{\prime }(\tilde{A})}\left(y\right)\rangle \;|\;y\in V\right\},\\ {\underline{R}}_{V}^{\prime }\left(\tilde{B}\right)=\left\{\langle x,\mu_{{\underline{R}}_{V}^{\prime }(\tilde{B})}\left(x\right),\nu_{{\underline{R}}_{V}^{\prime }(\tilde{B})}\left(x\right)\rangle \;|\;x\in U\right\},\\ {\underline{R}}_{V}^{\prime }\left(\tilde{B}\right)=\left\{\langle x,\mu_{{\underline{R}}_{V}^{\prime }(\tilde{B})}\left(x\right),\nu_{{\underline{R}}_{V}^{\prime }(\tilde{B})}\left(x\right)\rangle \;|\;x\in U\right\}, \end{array}$$

where

(23) μR_U′(A~)(y)=∨{α ∣ y∈R_U(A~αβ)}=∨{α ∣ Rp(y)⊆A~αβ},νR_U′(A~)(y)=∧{β ∣ y∈R_U(A~αβ)}=∧{β ∣ Rp(y)⊆A~αβ},μR¯U′(A~)(y)=∨{α ∣ y∈R¯U(A~αβ)}=∨{α ∣ Rp(y)∩A~αβ≠ϕ},νR¯U′(A~)(y)=∧{β ∣ y∈R¯U(A~αβ)}=∧{β ∣ Rp(y)∩A~αβ≠ϕ},μR_V′(B~)(x)=∨{α ∣ x∈R_V(B~αβ)}=∨{α ∣ Rs(x)⊆B~αβ},νR_V′(B~)(x)=∧{β ∣ x∈R_V(B~αβ)}=∧{β ∣ Rs(x)⊆B~αβ},μR¯V′(B~)(x)=∨{α ∣ x∈R¯V(B~αβ)}=∨{α ∣ Rs(x)∩B~αβ≠ϕ},νR¯V′(B~)(x)=∧{β ∣ x∈R¯V(B~αβ)}=∧{β ∣ Rs(x)∩B~αβ≠ϕ}.

Then, we can obtain the following properties.

**Theorem 16**

Let (*U*, *V*, *R*) be a generalized approximation space over two universes, and for any $\tilde{A}\in IF(U)$, $\tilde{B}\in IF(V)$, then

(24) $$\begin{array}{c} {\underline{R}}_{U}\left(\tilde{A}\right)={\underline{R}}_{U}^{\prime }\left(\tilde{A}\right),\;\;{\bar{R}}_{U}\left(\tilde{A}\right)={\bar{R}}_{U}^{\prime }\left(\tilde{A}\right);\\ {\underline{R}}_{V}\left(\tilde{B}\right)={\underline{R}}_{V}^{\prime }\left(\tilde{B}\right),\;\;{\bar{R}}_{V}\left(\tilde{B}\right)={\bar{R}}_{V}^{\prime }\left(\tilde{B}\right). \end{array}$$

**Proof**

For any *y* ∈ *V*, denote

(25) $$\begin{array}{c} \alpha_{1}=\mu_{{\underline{R}}_{U}(A)}\left(y\right)=\overset{}{\underset{x\in R_{p}(y)}{⋀}}\mu_{\tilde{A}}\left(x\right),\\ \beta_{1}=\nu_{{\underline{R}}_{U}(A)}\left(y\right)=\overset{}{\underset{x\in R_{p}(y)}{⋁}}\nu_{\tilde{A}}\left(x\right);\\ \alpha_{2}=\vee \left\{\alpha \;|\;R_{p}\left(y\right)\subseteq {\tilde{A}}_{\alpha }^{\beta }\right\},\;\;\beta_{2}=\wedge \left\{\beta \;|\;R_{p}\left(y\right)\subseteq {\tilde{A}}_{\alpha }^{\beta }\right\}. \end{array}$$

Let *α*, *β* satisfy $R_{p}(y)\subseteq {\tilde{A}}_{\alpha }^{\beta }$, if *x* ∈ *R*_*p*_(*y*), then $\mu_{\tilde{A}(x)}\geq \alpha$, $\nu_{\tilde{A}(x)}\leq \beta$ and ${⋀}_{x\in R_{p}(y)}^{}\mu_{\tilde{A}}(x)\geq \alpha$, ${⋁}_{x\in R_{p}(y)}^{}\nu_{\tilde{A}}(x)\leq \beta$. So, *α*_1_ ≥ *α*, *β*_1_ ≤ *β*; therefore; *α*_1_ ≥ *α*_2_, *β*_1_ ≤ *β*_2_.

On the other hand, for any *α* > *α*_2_, *β* < *β*_2_, according to the definitions of *α*_2_, *β*_2_, we can know that there exists *x* ∈ *R*_*p*_(*y*), s.t. $x\notin {\tilde{A}}_{\alpha }^{\beta }$; that is, $\alpha_{1}\leq \mu_{\tilde{A}}(x)<\alpha$, $\beta_{1}\geq \nu_{\tilde{A}}(x)>\beta$; thus, *α* > *α*_1_, *β* < *β*_1_, by the arbitrary of *α* > *α*_2_ and *β* < *β*_2_, and we can obtain *α*_2_ ≥ *α*_1_, *β*_2_ ≤ *β*_1_. Hence, ${\underline{R}}_{U}(\tilde{A})={\underline{R}}_{U}^{\prime }(\tilde{A})$.

The properties ${\bar{R}}_{U}(\tilde{A})={\bar{R}}_{U}^{\prime }(\tilde{A})$, ${\underline{R}}_{V}(\tilde{B})={\underline{R}}_{V}^{\prime }(\tilde{B})$, and ${\bar{R}}_{V}(\tilde{B})={\bar{R}}_{V}^{\prime }(\tilde{B})$ can be proved similarly.

**Definition 17**

Let (*U*, *V*, *R*) be a generalized approximation space over two universes, ${\tilde{A}}_{1},{\tilde{A}}_{2}\in IF(U)$, ${\tilde{B}}_{1},{\tilde{B}}_{2}\in IF(V)$.

 If ${\underline{R}}_{U}({\tilde{A}}_{1})={\underline{R}}_{U}({\tilde{A}}_{2})$, then ${\tilde{A}}_{1}$ and ${\tilde{A}}_{2}$ are called lower rough equivalences of *U*, denoted by ${\tilde{A}}_{1}{≂}_{U}{\tilde{A}}_{2}$.
 If ${\bar{R}}_{U}({\tilde{A}}_{1})={\bar{R}}_{U}({\tilde{A}}_{2})$, then ${\tilde{A}}_{1}$ and ${\tilde{A}}_{2}$ are called upper rough equivalences of *U*, denoted by ${\tilde{A}}_{1}{≃}_{U}{\tilde{A}}_{2}$.
 If ${\underline{R}}_{U}({\tilde{A}}_{1})={\underline{R}}_{U}({\tilde{A}}_{2})$ and ${\bar{R}}_{U}({\tilde{A}}_{1})={\bar{R}}_{U}({\tilde{A}}_{2})$, then ${\tilde{A}}_{1}$ and ${\tilde{A}}_{2}$ are called rough equivalences of *U*, denoted by ${\tilde{A}}_{1}\approx_{U}{\tilde{A}}_{2}$.
 If ${\underline{R}}_{V}({\tilde{B}}_{1})={\underline{R}}_{V}({\tilde{B}}_{2})$, then ${\tilde{B}}_{1}$ and ${\tilde{B}}_{2}$ are called lower rough equivalences of *V*, denoted by ${\tilde{B}}_{1}{≂}_{V}{\tilde{B}}_{2}$.
 If ${\bar{R}}_{V}({\tilde{B}}_{1})={\bar{R}}_{V}({\tilde{B}}_{2})$, then ${\tilde{B}}_{1}$ and ${\tilde{B}}_{2}$ are called upper rough equivalences of *V*, denoted by ${\tilde{B}}_{1}{≃}_{V}{\tilde{B}}_{2}$.
 If ${\underline{R}}_{V}({\tilde{B}}_{1})={\underline{R}}_{V}({\tilde{B}}_{2})$ and ${\bar{R}}_{V}({\tilde{B}}_{1})={\bar{R}}_{V}({\tilde{B}}_{2})$, then ${\tilde{B}}_{1}$ and ${\tilde{B}}_{2}$ are called rough equivalences of *V*, denoted by ${\tilde{B}}_{1}\approx_{V}{\tilde{B}}_{2}$.

**Proposition 18**

Let (*U*, *V*, *R*) be a generalized approximation space over two universes, ${\tilde{A}}_{1},{\tilde{A}}_{2},{\tilde{A}}_{1}^{\prime },{\tilde{A}}_{2}^{\prime }\in IF(U)$, ${\tilde{B}}_{1},{\tilde{B}}_{2},{\tilde{B}}_{1}^{\prime },{\tilde{B}}_{2}^{\prime }\in IF(V)$; then,

1. ${\tilde{A}}_{1}{≂}_{U}{\tilde{A}}_{2}\Leftrightarrow ({\tilde{A}}_{1}\cap {\tilde{A}}_{2}){≂}_{U}{\tilde{A}}_{2}$, $({\tilde{A}}_{1}\cap {\tilde{A}}_{2}){≂}_{U}{\tilde{A}}_{1}$ *;*
2. ${\tilde{B}}_{1}{≂}_{V}{\tilde{B}}_{2}\Leftrightarrow ({\tilde{B}}_{1}\cap {\tilde{B}}_{2}){≂}_{V}B_{2}$, $({\tilde{B}}_{1}\cap {\tilde{B}}_{2}){≂}_{V}{\tilde{B}}_{1}$.
3. ${\tilde{A}}_{1}{≃}_{U}{\tilde{A}}_{2}\Leftrightarrow ({\tilde{A}}_{1}\cup {\tilde{A}}_{2}){≃}_{U}{\tilde{A}}_{2}$, $({\tilde{A}}_{1}\cup A_{2}){≃}_{U}{\tilde{A}}_{1}$ *;*
4. ${\tilde{B}}_{1}{≃}_{V}{\tilde{B}}_{2}\Leftrightarrow ({\tilde{B}}_{1}\cup {\tilde{B}}_{2}){≃}_{V}{\tilde{B}}_{2}$, $({\tilde{B}}_{1}\cup {\tilde{B}}_{2}){≃}_{V}{\tilde{B}}_{1}$.
5. * If* ${\tilde{A}}_{1}{≂}_{U}{\tilde{A}}_{1}^{\prime }$, ${\tilde{A}}_{2}{≂}_{U}{\tilde{A}}_{2}^{\prime }$ *, then* $({\tilde{A}}_{1}\cap {\tilde{A}}_{2}){≂}_{U}{\tilde{A}}_{1}^{\prime }\cap {\tilde{A}}_{2}^{\prime }$ *;*
6. *if* ${\tilde{A}}_{1}{≃}_{U}{\tilde{A}}_{1}^{\prime }$, ${\tilde{A}}_{2}{≃}_{U}{\tilde{A}}_{2}^{\prime }$ *, then* $({\tilde{A}}_{1}\cup {\tilde{A}}_{2}){≃}_{U}{\tilde{A}}_{1}^{\prime }\cup {\tilde{A}}_{2}^{\prime }$ *;*
7. *if* ${\tilde{B}}_{1}{≂}_{V}{\tilde{B}}_{1}^{\prime }$, ${\tilde{B}}_{2}{≂}_{V}{\tilde{B}}_{2}^{\prime }$ *, then* $({\tilde{B}}_{1}\cap {\tilde{B}}_{2}){≂}_{V}{\tilde{B}}_{1}^{\prime }\cap {\tilde{B}}_{2}^{\prime }$ *;*
8. *if* ${\tilde{B}}_{1}{≃}_{V}{\tilde{B}}_{1}^{\prime }$, ${\tilde{B}}_{2}{≃}_{V}{\tilde{B}}_{2}^{\prime }$ *, then* $({\tilde{B}}_{1}\cup {\tilde{B}}_{2}){≃}_{V}{\tilde{B}}_{1}^{\prime }\cup {\tilde{B}}_{2}^{\prime }$.
9. * If* ${\tilde{A}}_{1}{≂}_{U}\emptyset$ *or* ${\tilde{A}}_{1}^{\prime }{≂}_{U}\emptyset$ *, then* ${\tilde{A}}_{1}\cap {\tilde{A}}_{1}^{\prime }{≂}_{U}\emptyset$ *;*
10. *if* ${\tilde{B}}_{1}{≂}_{V}\emptyset$ *or* ${\tilde{B}}_{1}^{\prime }{≂}_{V}\emptyset$ *, then* ${\tilde{B}}_{1}\cap {\tilde{B}}_{1}^{\prime }{≂}_{V}\emptyset$.
11. * If* ${\tilde{A}}_{1}{≃}_{U}U$ *or* ${\tilde{A}}_{1}^{\prime }{≃}_{U}U$ *, then* ${\tilde{A}}_{1}\cup {\tilde{A}}_{1}^{\prime }{≃}_{U}U$ *;*
12. *if* ${\tilde{B}}_{1}{≃}_{V}V$ *or* ${\tilde{B}}_{1}^{\prime }{≃}_{V}V$ *, then* ${\tilde{B}}_{1}\cup {\tilde{B}}_{1}^{\prime }{≃}_{V}V$.
13. * If* ${\tilde{A}}_{1}\subseteq {\tilde{A}}_{1}^{\prime }$ *and* ${\tilde{A}}_{1}^{\prime }{≂}_{U}\emptyset$ *, then* ${\tilde{A}}_{1}{≂}_{U}\emptyset$ *;*
14. *if* ${\tilde{B}}_{1}\subseteq {\tilde{B}}_{1}^{\prime }$ *and* ${\tilde{B}}_{1}^{\prime }{≂}_{V}\emptyset$ *, then* ${\tilde{B}}_{1}{≂}_{V}\emptyset$.
15. * If* ${\tilde{A}}_{1}\subseteq {\tilde{A}}_{1}^{\prime }$ *and A*_1_≃_*U*_ *U, then* ${\tilde{A}}_{1}^{\prime }{≃}_{U}U$ *;*
16. *if* ${\tilde{B}}_{1}\subseteq {\tilde{B}}_{1}^{\prime }$ *and* ${\tilde{B}}_{1}{≃}_{V}V$ *, then* ${\tilde{B}}_{1}^{\prime }{≃}_{V}V$.

**Proof**

Straightforward.

**Theorem 19**

Let (*U*, *V*, *R*) be a generalized approximation space over two universes, $\tilde{A}\in IF(U)$, $\tilde{B}\in IF(V)$; then,

1. ${\underline{R}}_{U}(\tilde{A})=\cap \;\{{\tilde{A}}^{\prime }\in IF(U)|\tilde{A}{≂}_{U}{\tilde{A}}^{\prime }\}$, ${\underline{R}}_{V}(\tilde{B})=\cap \;\{{\tilde{B}}^{\prime }\in IF(V)|\tilde{B}{≂}_{V}{\tilde{B}}^{\prime }\}$;
2. ${\bar{R}}_{U}(\tilde{A})=\cup \;\{{\tilde{A}}^{\prime }\in IF(U)|\tilde{A}{≃}_{U}{\tilde{A}}^{\prime }\}$, ${\bar{R}}_{V}(\tilde{B})=\cup \;\{{\tilde{B}}^{\prime }\in IF(V)|\tilde{B}{≃}_{V}{\tilde{B}}^{\prime }\}$.

**Proof**

We can obtain them according to Proposition 18.

**Theorem 20**

Let (*U*, *V*, *R*) be a generalized approximation space over two universes, $\tilde{A}\in IF(U)$, for any 0 ≤ *α*, *α*_1_, *α*_2_, *β*, *β*_1_, *β*_2_ ≤ 1, and if *R* is a reverse serial relation on *U* × *V*, denote

(26) $$\begin{array}{c} {\left({\underline{R}}_{U}\left(\tilde{A}\right)\right)}_{\alpha }^{\beta }=\left\{y\in V\;|\;\mu_{{\underline{R}}_{U}(\tilde{A})}\left(y\right)\geq \alpha ,\nu_{{\underline{R}}_{U}(\tilde{A})}\left(y\right)\leq \beta \right\},\\ {\left({\bar{R}}_{U}\left(\tilde{A}\right)\right)}_{\alpha }^{\beta }=\left\{y\in V\mu_{{\bar{R}}_{U}(\tilde{A})}\left(y\right)\geq \alpha ,\nu_{{\bar{R}}_{U}(\tilde{A})}\left(y\right)\leq \beta \right\}. \end{array}$$

Then,

1. ${\left({\underline{R}}_{U}(\tilde{A})\right)}_{\alpha }^{\beta }⊇{\underline{R}}_{U}({\left(\tilde{A}\right)}_{\alpha }^{\beta })$, ${\left({\bar{R}}_{U}(\tilde{A})\right)}_{\alpha }^{\beta }⊇{\bar{R}}_{U}({\left(\tilde{A}\right)}_{\alpha }^{\beta })$,
2. *α*_1_ ≥ *α*_2_, $\beta_{1}\leq \beta_{2}\Rightarrow {\left({\underline{R}}_{U}(\tilde{A})\right)}_{\alpha_{1}}^{\beta_{1}}\subseteq {\left({\bar{R}}_{U}(\tilde{A})\right)}_{\alpha_{1}}^{\beta_{1}}\subseteq {\left({\bar{R}}_{U}(\tilde{A})\right)}_{\alpha_{2}}^{\beta_{2}}$.

**Proof**

(1) For any $y\in {\underline{R}}_{U}({\left(\tilde{A}\right)}_{\alpha }^{\beta })\Rightarrow \phi \neq R_{p}(y)\subseteq {\left(\tilde{A}\right)}_{\alpha }^{\beta }\Rightarrow$ for any $x\in R_{p}(y)\subseteq {\left(\tilde{A}\right)}_{\alpha }^{\beta }\Rightarrow \text{for}\;\text{all}\;x\in R_{p}(y)$, $\mu_{\tilde{A}}(x)\geq \alpha$, $\nu_{\tilde{A}}(x)\leq \beta \Rightarrow {⋀}_{x\in R_{p}(y)}^{}\mu_{\tilde{A}}(x)\geq \alpha$, ${⋁}_{x\in R_{p}(y)}^{}\nu_{\tilde{A}}(x)\leq \beta \Rightarrow \mu_{{\underline{R}}_{U}(\tilde{A})}(y)\geq \alpha$, and $\nu_{{\underline{R}}_{U}(\tilde{A})}(y)\leq \beta \Rightarrow y\in {\left({\underline{R}}_{U}(\tilde{A})\right)}_{\alpha }^{\beta }$. Thus, we can have ${\left({\underline{R}}_{U}(\tilde{A})\right)}_{\alpha }^{\beta }⊇{\underline{R}}_{U}({\left(\tilde{A}\right)}_{\alpha }^{\beta })$.

The properties ${\left({\bar{R}}_{U}(\tilde{A})\right)}_{\alpha }^{\beta }⊇{\bar{R}}_{U}({\left(\tilde{A}\right)}_{\alpha }^{\beta })$ can be proved similarly.

(2) Since *α*_1_ ≥ *α*_2_, *β*_1_ ≤ *β*_2_, we can have that for any $y\in {\left({\underline{R}}_{U}(\tilde{A})\right)}_{\alpha_{1}}^{\beta_{1}}\Rightarrow \mu_{{\underline{R}}_{U}(\tilde{A})}(y)={⋀}_{x\in R_{p}(y)}^{}\mu_{\tilde{A}}(x)\geq \alpha_{1}$, $\nu_{{\underline{R}}_{U}(\tilde{A})}(y)={⋁}_{x\in R_{p}(y)}^{}\nu_{\tilde{A}}(x)\leq \beta_{1}\Rightarrow {⋁}_{x\in R_{p}(y)}^{}\mu_{\tilde{A}}(x)\geq \alpha_{1}\geq \alpha_{2}$, and ${⋀}_{x\in R_{p}(y)}^{}\nu_{\tilde{A}}(x)\leq \beta_{1}\leq \beta_{2}\Rightarrow y\in {\left({\bar{R}}_{U}(\tilde{A})\right)}_{\alpha_{2}}^{\beta_{2}}$. Therefore, ${\left({\underline{R}}_{U}(\tilde{A})\right)}_{\alpha_{1}}^{\beta_{1}}\subseteq {\left({\bar{R}}_{U}(\tilde{A})\right)}_{\alpha_{1}}^{\beta_{1}}\subseteq {\left({\bar{R}}_{U}(\tilde{A})\right)}_{\alpha_{2}}^{\beta_{2}}$.

**Theorem 21**

Let (*U*, *V*, *R*) be a generalized approximation space over two universes, $\tilde{A}\in IF(U)$, for any 0 ≤ *α*, *α*_1_, *α*_2_, *β*, *β*_1_, *β*_2_ ≤ 1, and if *R* is a serial relation on *U* × *V*, denote

(27) $$\begin{array}{c} {\left({\underline{R}}_{V}\left(\tilde{B}\right)\right)}_{\alpha }^{\beta }=\left\{x\in V\;|\;\mu_{{\underline{R}}_{V}(\tilde{B})}\left(x\right)\geq \alpha ,\nu_{{\underline{R}}_{V}(\tilde{B})}\left(x\right)\leq \beta \right\},\\ {\left({\bar{R}}_{V}\left(\tilde{B}\right)\right)}_{\alpha }^{\beta }=\left\{x\in V\mu_{{\bar{R}}_{V}(\tilde{B})}\left(x\right)\geq \alpha ,\nu_{{\bar{R}}_{V}(\tilde{B})}\left(x\right)\leq \beta \right\}. \end{array}$$

Then,

1. ${\left({\underline{R}}_{V}(\tilde{B})\right)}_{\alpha }^{\beta }⊇{\underline{R}}_{V}({\left(\tilde{B}\right)}_{\alpha }^{\beta })$, ${\left({\bar{R}}_{V}(\tilde{B})\right)}_{\alpha }^{\beta }⊇{\bar{R}}_{V}({\left(\tilde{B}\right)}_{\alpha }^{\beta })$,
2. *α*_1_ ≥ *α*_2_, $\beta_{1}\leq \beta_{2}\Rightarrow {\left({\underline{R}}_{V}(\tilde{B})\right)}_{\alpha_{1}}^{\beta_{1}}\subseteq {\left({\bar{R}}_{V}(\tilde{B})\right)}_{\alpha_{1}}^{\beta_{1}}\subseteq {\left({\bar{R}}_{V}(\tilde{B})\right)}_{\alpha_{2}}^{\beta_{2}}$.

**Proof**

The proof is similar to Theorem 20.

## 4. The Measures of Rough Atanassov's Intuitionistic Fuzzy Sets Model over Two Universes

In this section, we will research some measures of rough Atanassov's intuitionistic fuzzy set over different universes.

**Definition 22**

Let (*U*, *V*, *R*) be a generalized approximation space over two universes, $\tilde{A}\in IF(U)$, for any 0 < *α*_2_ ≤ *α*_1_ ≤ 1, 0 < *β*_1_ ≤ *β*_2_ ≤ 1, and the approximate precision of $\tilde{A}$ about *R* can be defined as follows:

(28) $$\begin{array}{c} \alpha_{U}{\left(\tilde{A}\right)}_{\left[(\alpha_{1},\beta_{1}),(\alpha_{2},\beta_{2})\right]}=\frac{\left|{\left({\underline{R}}_{U}\left(\tilde{A}\right)\right)}_{\alpha_{1}}^{\beta_{1}}\right|}{\left|{\left({\bar{R}}_{U}\left(\tilde{A}\right)\right)}_{\alpha_{2}}^{\beta_{2}}\right|}, \end{array}$$

where $\tilde{A}\neq \emptyset$ and the notation |·| denotes the cardinality of set.

Let $\rho_{U}{\left(\tilde{A}\right)}_{\left[(\alpha_{1},\beta_{1}),(\alpha_{2},\beta_{2})\right]}=1-\alpha_{U}{\left(\tilde{A}\right)}_{\left[(\alpha_{1},\beta_{1}),(\alpha_{2},\beta_{2})\right]}$, and $\rho_{U}{\left(\tilde{A}\right)}_{\left[(\alpha_{1},\beta_{1}),(\alpha_{2},\beta_{2})\right]}$ is called the rough degree of $\tilde{A}$ about the universe *U*.

**Theorem 23**

Let (*U*, *V*, *R*) be a generalized approximation space over two universes, $\tilde{A}\in IF(U)$, for any 0 < *α*_2_ ≤ *α*_1_ ≤ 1, 0 < *β*_1_ ≤ *β*_2_ ≤ 1, and the approximate precision $\alpha_{U}{\left(\tilde{A}\right)}_{\left[(\alpha_{1},\beta_{1}),(\alpha_{2},\beta_{2})\right]}$ and the rough degree $\rho_{U}{\left(\tilde{A}\right)}_{\left[(\alpha_{1},\beta_{1}),(\alpha_{2},\beta_{2})\right]}$ satisfy the properties as follows:

(29) $$\begin{array}{c} 0\leq \alpha_{U}{\left(\tilde{A}\right)}_{\left[\left(\alpha_{1},\beta_{1}),(\alpha_{2},\beta_{2}\right)\right]}\leq 1,\\ 0\leq \rho_{U}{\left(\tilde{A}\right)}_{\left[\left(\alpha_{1},\beta_{1}),(\alpha_{2},\beta_{2}\right)\right]}\leq 1. \end{array}$$

**Proof**

According to Definition 22, this theorem can be proved easily.

**Example 24 (continued from Example 8)**

We can find the (0.8,0.1)-level cut set of ${\underline{R}}_{U}(\tilde{A})$ and the (0.6,0.1)-level cut set of $\bar{R}(\tilde{A})$ as follows:

(30) $$\begin{array}{c} {\left({\underline{R}}_{U}\left(\tilde{A}\right)\right)}_{0.8}^{0.1}=\emptyset ,\;\;{\left({\bar{R}}_{U}\left(\tilde{A}\right)\right)}_{0.6}^{0.1}=\left\{y_{1},y_{2},y_{3}\right\}. \end{array}$$

So, we can compute the approximation precision and rough degree as follows:

(31) $$\begin{array}{c} \alpha_{U}^{R}{\left(\tilde{A}\right)}_{\left[(0.8,0.1),(0.6,0.1)\right]}=0,\;\;\rho_{U}^{R}{\left(\tilde{A}\right)}_{\left[(0.8,0.1),(0.6,0.1)\right]}=1. \end{array}$$

**Theorem 25**

Let (*U*, *V*, *R*) be a generalized approximation space over two universes, $\tilde{A}$, ${\tilde{A}}_{1}\in IF(U)$, $\tilde{A}\subseteq {\tilde{A}}_{1}$, and ${\left({\bar{R}}_{U}A\right)}_{\alpha_{2}}^{\beta_{2}}={\left({\bar{R}}_{U}A_{1}\right)}_{\alpha_{2}}^{\beta_{2}}$, for any 0 < *α*_2_ ≤ *α*_1_ ≤ 1, 0 < *β*_1_ ≤ *β*_2_ ≤ 1; then,

(32) $$\begin{array}{c} \alpha_{U}{\left(\tilde{A}\right)}_{\left[(\alpha_{1},\beta_{1}),(\alpha_{2},\beta_{2})\right]}\leq \alpha_{U}{\left({\tilde{A}}_{1}\right)}_{\left[(\alpha_{1},\beta_{1}),(\alpha_{2},\beta_{2})\right]},\\ \rho_{U}{\left({\tilde{A}}_{1}\right)}_{\left[(\alpha_{1},\beta_{1}),(\alpha_{2},\beta_{2})\right]}\leq \rho_{U}{\left(\tilde{A}\right)}_{\left[(\alpha_{1},\beta_{1}),(\alpha_{2},\beta_{2})\right]}. \end{array}$$

**Proof**

Since $\tilde{A}\subseteq {\tilde{A}}_{1}$, we can have ${\left({\underline{R}}_{U}(\tilde{A})\right)}_{\alpha_{1}}^{\beta_{1}}\subseteq {\left({\underline{R}}_{U}({\tilde{A}}_{1})\right)}_{\alpha_{1}}^{\beta_{1}}$. On the other hand, ${\left({\bar{R}}_{U}(\tilde{A})\right)}_{\alpha_{2}}^{\beta_{2}}={\left({\bar{R}}_{U}({\tilde{A}}_{1})\right)}_{\alpha_{2}}^{\beta_{2}}$. Therefore, the theorem can be proved by Definition 22.

**Theorem 26**

Let (*U*, *V*, *R*) be a generalized approximation space over two universes, $\tilde{A},{\tilde{A}}_{1}\in IF(U)$, $\tilde{A}\subseteq {\tilde{A}}_{1}$, ${\left({\underline{R}}_{U}(\tilde{A})\right)}_{\alpha_{1}}^{\beta_{1}}={\left({\underline{R}}_{U}({\tilde{A}}_{1})\right)}_{\alpha_{2}}^{\beta_{2}}$, for any 0 < *α*_2_ ≤ *α*_1_ ≤ 1, 0 < *β*_1_ ≤ *β*_2_ ≤ 1; then,

(33) $$\begin{array}{c} \alpha_{U}{\left({\tilde{A}}_{1}\right)}_{\left[(\alpha_{1},\beta_{1}),(\alpha_{2},\beta_{2})\right]}\leq \alpha_{U}{\left(\tilde{A}\right)}_{\left[(\alpha_{1},\beta_{1}),(\alpha_{2},\beta_{2})\right]},\\ \rho_{U}{\left(\tilde{A}\right)}_{\left[(\alpha_{1},\beta_{1}),(\alpha_{2},\beta_{2})\right]}\leq \rho_{U}{\left({\tilde{A}}_{1}\right)}_{\left[(\alpha_{1},\beta_{1}),(\alpha_{2},\beta_{2})\right]}. \end{array}$$

**Proof**

The proof is similar to Theorem 25.

**Theorem 27**

Let $\tilde{A},{\tilde{A}}_{1}\in F(U)$, and if ${\tilde{A}}_{1}\approx_{U}\tilde{A}$, for any 0 < *α*_2_ ≤ *α*_1_ ≤ 1, 0 < *β*_1_ ≤ *β*_2_ ≤ 1, one can have

(34) $$\begin{array}{c} \alpha_{U}{\left({\tilde{A}}_{1}\right)}_{\left[(\alpha_{1},\beta_{1}),(\alpha_{2},\beta_{2})\right]}=\alpha_{U}{\left(\tilde{A}\right)}_{\left[(\alpha_{1},\beta_{1}),(\alpha_{2},\beta_{2})\right]},\\ \rho_{U}{\left(\tilde{A}\right)}_{\left[(\alpha_{1},\beta_{1}),(\alpha_{2},\beta_{2})\right]}=\rho_{U}{\left({\tilde{A}}_{1}\right)}_{\left[(\alpha_{1},\beta_{1}),(\alpha_{2},\beta_{2})\right]}. \end{array}$$

**Proof**

It can be proved by Theorem 25 and Definition 22.

**Theorem 28**

Let *U*, *V* be two nonempty finite universes, and let *R* be the relation of  *U* × *V*. For any $\tilde{A}$, ${\tilde{A}}_{1}\in IF(U)$. The rough degrees and precisions of $\tilde{A}$, ${\tilde{A}}_{1}$, $\tilde{A}\cup {\tilde{A}}_{1}$, and $\tilde{A}\cap {\tilde{A}}_{1}$ have the following relations. Consider

(35) ρU(A~∪A~1)[(α1,β1),(α2,β2)]|(R¯U(A~))α2β2∪(R¯U(A~1))α2β2| ≤ρU(A~)[(α1,β1),(α2,β2)]|(R¯U(A~))α2β2|  +ρU(A~1)[(α1,β1),(α2,β2)]|(R¯U(A~1))α2β2|  −ρU(A~∩A~1)[(α1,β1),(α2,β2)]  ×|(R¯U(A~))α2β2∩(R¯U(A~1))α2β2|,αU(A~∪A~1)[(α1,β1),(α2,β2)]|(R¯U(A~))α2β2∪(R¯U(A~1))α2β2| ≥αU(A~)[(α1,β1),(α2,β2)]|(R¯U(A~))α2β2|  +αU(A~1)[(α1,β1),(α2,β2)]|(R¯U(A~1))α2β2|  −αU(A~∩A~1)[(α1,β1),(α2,β2)]  ×|(R¯U(A~))α2β2∩(R¯U(A~1))α2β2|.

**Proof**

According to Theorem 10, we can obtain

(36) ρU(A~∪A~1)[(α1,β1),(α2,β2)]=1−|(R_U(A~∪A~1))α1β1||(R¯U(A~∪A~1))α2β2|=1−|(R_U(A~∪A~1))α1β1||(R¯U(A~))α2β2∪(R¯U(A~1))α2β2|≤1−|(R_U(A~))α1β1∪(R_U(A~1))α1β1||(R¯U(A~))α2β2∪(R¯U(A~1))α2β2|.

On the other hand,

(37) ρU(A~∩A~1)[(α1,β1),(α2,β2)]=1−|(R_U(A~∩A~1))α1β1||(R¯U(A~∩A~1))α2,β2|=1−|(R_U(A~))α1β1∩(R_U(A~1))α1β1||(R¯U(A~∪A~1))α2β2|≤1−|(R_U(A~))α1β1∩(R_U(A~1))α1β1||(R¯U(A~))α2β2∩(R¯U(A~1))α2β2|.

For classical sets *X* and *Y*, we have

(38) $$\begin{array}{c} \left|X\cup Y\right|=\left|X\right|+\left|Y\right|-\left|X\cap Y\right|. \end{array}$$

Hence,

(39) ρU(A~∪A~1)[(α1,β1),(α2,β2)]|(R¯U(A~))α2β2∪(R¯U(A~1))α2β2|≤|(R¯U(A~))α2β2∪(R¯U(A~1))α2β2| −|(R_U(A~))α1β1∪(R_U(A~1))α1β1|=|(R¯U(A~))α2β2|+|(R¯U(A~1))α2β2| −|(R¯U(A~))α2β2∩(R¯U(A~1))α2β2| −|(R_U(A~))α1β1|−|(R_U(A~1))α1β1| +|(R_U(A~))α1β1∩(R_U(A~1))α2β2|≤|(R¯U(A~))α2β2|+|(R¯U(A~1))α2β2|−|(R_U(A~))α1β1| −|(R_U(A~1))α1β1| −ρU(A~∩A~1)[(α1,β1),(α2,β2)]|(R¯U(A~))α2β2∩(R¯U(A~1))α2β2|=ρU(A~)[(α1,β1),(α2,β2)]|(R¯U(A~))α2β2| +ρU(A~1)[(α1,β1),(α2,β2)]|(R¯U(A~1))α2β2| −ρU(A~∩A~1)[(α1,β1),(α2,β2)]|(R¯U(A~))α2β2∩(R¯U(A~1))α2β2|.

The other inequality can be proved similarly.

**Proposition 29**

Let (*U*, *V*, *R*), (*U*, *V*, *S*) be two generalized approximation spaces over two universes, *S*⊆*R*,

(40) $$\begin{array}{c} \alpha_{U}^{R}{\left(\tilde{A}\right)}_{\left[(\alpha_{1},\beta_{1}),(\alpha_{2},\beta_{2})\right]}=\frac{\left|{\left({\underline{R}}_{U}\tilde{A}\right)}_{\alpha_{1}}^{\beta_{1}}\right|}{\left|{\left({\bar{R}}_{U}A\right)}_{\alpha_{2}}^{\beta_{2}}\right|},\\ \alpha_{U}^{S}{\left(\tilde{A}\right)}_{\left[(\alpha_{1},\beta_{1}),(\alpha_{2},\beta_{2})\right]}=\frac{\left|{\left({\underline{R}}_{U}\tilde{A}\right)}_{\alpha_{1}}^{\beta_{1}}\right|}{\left|{\left({\bar{R}}_{U}A\right)}_{\alpha_{2}}^{\beta_{2}}\right|},\\ \rho_{U}^{R}{\left(\tilde{A}\right)}_{\left[(\alpha_{1},\beta_{1}),(\alpha_{2},\beta_{2})\right]}=1-\frac{\left|{\left({\underline{R}}_{U}\tilde{A}\right)}_{\alpha_{1}}^{\beta_{1}}\right|}{\left|{\left({\bar{R}}_{U}A\right)}_{\alpha_{2}}^{\beta_{2}}\right|},\\ \rho_{U}^{S}{\left(\tilde{A}\right)}_{\left[(\alpha_{1},\beta_{1}),(\alpha_{2},\beta_{2})\right]}=1-\frac{\left|{\left({\underline{R}}_{U}\tilde{A}\right)}_{\alpha_{1}}^{\beta_{1}}\right|}{\left|{\left({\bar{R}}_{U}A\right)}_{\alpha_{2}}^{\beta_{2}}\right|},\\ \alpha_{V}^{R}{\left(\tilde{B}\right)}_{\left[(\alpha_{1},\beta_{1}),(\alpha_{2},\beta_{2})\right]}=\frac{\left|{\left({\underline{R}}_{V}\tilde{B}\right)}_{\alpha_{1}}^{\beta_{1}}\right|}{\left|{\left({\bar{R}}_{V}B\right)}_{\alpha_{2}}^{\beta_{2}}\right|},\\ \alpha_{V}^{S}{\left(\tilde{B}\right)}_{\left[(\alpha_{1},\beta_{1}),(\alpha_{2},\beta_{2})\right]}=\frac{\left|{\left({\underline{R}}_{V}\tilde{B}\right)}_{\alpha_{1}}^{\beta_{1}}\right|}{\left|{\left({\bar{R}}_{V}B\right)}_{\alpha_{2}}^{\beta_{2}}\right|},\\ \rho_{V}^{R}{\left(\tilde{B}\right)}_{\left[(\alpha_{1},\beta_{1}),(\alpha_{2},\beta_{2})\right]}=1-\frac{\left|{\left({\underline{R}}_{V}\tilde{B}\right)}_{\alpha_{1}}^{\beta_{1}}\right|}{\left|{\left({\bar{R}}_{V}B\right)}_{\alpha_{2}}^{\beta_{2}}\right|},\\ \rho_{V}^{S}{\left(\tilde{B}\right)}_{\left[(\alpha_{1},\beta_{1}),(\alpha_{2},\beta_{2})\right]}=1-\frac{\left|{\left({\underline{R}}_{V}\tilde{B}\right)}_{\alpha_{1}}^{\beta_{1}}\right|}{\left|{\left({\bar{R}}_{V}B\right)}_{\alpha_{2}}^{\beta_{2}}\right|}. \end{array}$$

Then, we can obtain

(41) $$\begin{array}{c} \alpha_{U}^{R}{\left(\tilde{A}\right)}_{\left[(\alpha_{1},\beta_{1}),(\alpha_{2},\beta_{2})\right]}\leq \alpha_{U}^{S}{\left(\tilde{A}\right)}_{\left[(\alpha_{1},\beta_{1}),(\alpha_{2},\beta_{2})\right]},\\ \rho_{U}^{S}{\left(\tilde{A}\right)}_{\left[(\alpha_{1},\beta_{1}),(\alpha_{2},\beta_{2})\right]}\leq \rho_{U}^{R}{\left(\tilde{A}\right)}_{\left[(\alpha_{1},\beta_{1}),(\alpha_{2},\beta_{2})\right]},\\ \alpha_{V}^{R}{\left(\tilde{B}\right)}_{\left[(\alpha_{1},\beta_{1}),(\alpha_{2},\beta_{2})\right]}\leq \alpha_{V}^{S}{\left(\tilde{B}\right)}_{\left[(\alpha_{1},\beta_{1}),(\alpha_{2},\beta_{2})\right]},\\ \rho_{V}^{S}{\left(\tilde{B}\right)}_{\left[(\alpha_{1},\beta_{1}),(\alpha_{2},\beta_{2})\right]}\leq \rho_{V}^{R}{\left(\tilde{B}\right)}_{\left[(\alpha_{1},\beta_{1}),(\alpha_{2},\beta_{2})\right]}. \end{array}$$

**Example 30 (continued from Examples 12 and 24)**

In the following, we give another binary relation *S* over the universes *U* and *V*

(42) $$\begin{array}{c} S=\left\{\left(x_{1},y_{2}\right),\left(x_{2},y_{3}\right),\left(x_{3},y_{4}\right),\left(x_{4},y_{1}\right),\left(x_{5},y_{5}\right)\right\}. \end{array}$$

Obviously, *S*⊆*R*, and by Definition 7, we can obtain

(43) S_U(A~)={〈y1,0.6,0.1〉, 〈y2,0.8,0.1〉, 〈y3,0.6,0.1〉,〈y4,0.2,0.8〉, 〈y5,0.1,0.6〉},S¯U(A~)={〈y1,0.6,0.1〉, 〈y2,0.8,0.1〉, 〈y3,0.6,0.1〉,〈y4,0.2,0.8〉, 〈y5,0.1,0.6〉}.

At this time,

(44) $$\begin{array}{c} {\left({\underline{S}}_{U}\left(\tilde{A}\right)\right)}_{0.8}^{0.1}=\left\{y_{2}\right\},\;\;{\left({\bar{S}}_{U}\left(\tilde{A}\right)\right)}_{0.6}^{0.1}=\left\{y_{1},y_{2},y_{3}\right\}. \end{array}$$

Then,

(45) $$\begin{array}{c} \alpha_{U}^{S}{\left(\tilde{A}\right)}_{\left[(0.8,0.1),(0.6,0.1)\right]}=\frac{1}{3},\;\;\rho_{U}^{S}{\left(\tilde{A}\right)}_{\left[(0.8,0.1),(0.6,0.1)\right]}=\frac{2}{3}. \end{array}$$

So,

(46) $$\begin{array}{c} \alpha_{U}^{R}{\left(\tilde{A}\right)}_{\left[(0.8,0.1),(0.6,0.1)\right]}\leq \alpha_{U}^{S}{\left(\tilde{A}\right)}_{\left[(0.8,0.1),(0.6,0.1)\right]},\\ \rho_{U}^{S}{\left(\tilde{A}\right)}_{\left[(0.8,0.1),(0.6,0.1)\right]}\leq \rho_{U}^{R}{\left(\tilde{A}\right)}_{\left[(0.8,0.1),(0.6,0.1)\right]}. \end{array}$$

Similarly, we can discuss that the approximated set is Atanassov's intuitionistic fuzzy set over the universe *V*.

## 5. Case Study

In medical diagnosis, sometimes we need to arrange patients' order according to the state of their illnesses. If the patient is in a bad way, he or she should be arranged to see a doctor first. If the illness is lighter, we can let him or her wait for the time being. So, a good ordering system is needed. When the patients' symptoms are Atanassov's intuitionistic fuzzy sets and the relationship between the symptoms and diseases is a general binary relation decided by medical specialists who have abundant experience, now, we will design the order system in view of the actual situation of the hospital by using rough Atanassov's intuitionistic fuzzy sets theory in order to consider most of the patients. This scheme can be used in both primary diagnosis and further diagnosis. Assume triple (*U*, *V*, *R*) is a generalized approximation space, where *U* is a symptom set, *V* is a disease set, and *R* is a relation from *U* to *V*. In general, the relation *R* can be given by medical specialist. Detailed steps are devised as follows.

**Step 1**

Weight Atanassov's intuitionistic fuzzy sets of patients' symptoms.

Because some symptoms are severe and some symptoms are not severe, we can weigh the symptoms in order to know the overall situation of the patient. Let $\tilde{A}=\{\langle x_{1},\mu_{\tilde{A}}(x_{1}),\nu_{\tilde{A}}(x_{1})\rangle$, $\langle x_{2},\mu_{\tilde{A}}(x_{2}),\nu_{\tilde{A}}(x_{2})\rangle ,\ldots ,\langle x_{n},\mu_{\tilde{A}}(x_{n}),\nu_{\tilde{A}}(x_{n})\rangle \}$ be Atanassov's intuitionistic fuzzy set of a patient, and ley *ω* = (*ω*_1_, *ω*_2_,…, *ω*_*n*_) be a weighting vector satisfying *ω*_1_ + *ω*_2_ + ⋯+*ω*_*n*_ = 1. The weighted intuitionistic fuzzy set of a patient can be obtained as follows:

(47) A~ω={〈x1,1−(1−μA~(x1))nω1,(νA~(x1))nω1〉,〈x2,1−(1−μA~(x2))nω2,(νA~(x2))nω1〉,…,〈xn,1−(1−μA~(xn))nωn,(νA~(xn))nωn〉}.

**Step 2**

Compute the lower and upper approximations of weighted Atanassov's intuitionistic fuzzy sets of patients' symptoms according to the model we proposed in Definition 7.

**Step 3**

Weigh the lower and upper approximations of Atanassov's intuitionistic fuzzy sets.

**Step 4**

By using Atanassov's intuitionistic fuzzy hybrid operator [30], calculate weighted lower and upper approximations of Atanassov's intuitionistic fuzzy sets' comprehensive attribute value *d*, and the weighting vector *w* = (*w*_1_, *w*_2_,…, *w*_*n*_) that satisfied *w*_1_ + *w*_2_ + ⋯+*w*_*n*_ = 1 is usually given by medical specialist in advance because objects of the lower and upper approximations of weighted Atanassov's intuitionistic fuzzy sets are subsets of disease sets. If the patients know the conditions of themselves well, we can consider the attribute value in terms of the lower approximations. If the patients are not familiar with conditions of themselves well, we can consider the attribute value in terms of the upper approximations. The lower and upper approximation comprehensive attribute values are defined as follows, respectively. Consider

(48) d(R_U(A~ω))=IFHw((μR_U(A~ω)(y1),νR_U(A~ω)(y1)),(μR_U(A~ω)(y2),νR_U(A~ω)(y2)),…,(μR_U(A~ω)(yn),νR_U(A~ω)(yn)))=(1−∏i=1n(1−μR_U(A~ω)(yi))nwi,∏i=1n(νR_U(A~ω)(yi))nwi),d(R¯U(A~ω))=IFHw((μR¯U(A~ω)(y1),νR¯U(A~ω)(y1)),(μR¯U(A~ω)(y2),νR¯U(A~ω)(y2)),…,(μR¯U(A~ω)(yn),νR¯U(A~ω)(yn)))=(1−∏i=1n(1−μR¯U(A~ω)(yi))nwi,∏i=1n(νR¯U(A~ω)(yi))nwi).

**Step 5**

Compute the comprehensive score value *s*. The higher the score, the more serious the patient. Then, we can order the patients in the view of comprehensive score values. According to Step 4, we also can gain two types of comprehensive score values, the first type of the comprehensive score value is about the lower approximation and the second type of the comprehensive score value is about the upper approximation. The results of comprehensive score values are shown as follows:

(49) s(R_U(A~ω))=(1−∏i=1n(1−μR_U(A~ω)(yi))nwi)−(∏i=1n(νR_U(A~ω)(yi))nwi),s(R¯U(A~ω))=(1−∏i=1n(1−μR¯U(A~ω)(yi))nwi)−(∏i=1n(νR¯U(A~ω)(yi))nwi).

Finally, we can order the patients to see the doctor in turn.

**Example**

Let us consider the problem about how to arrange patients for a hospital. These patients have enough time and they agree with the project which mainly considers the conditions of patients. That is to say, the more severe the patient is, the earlier he can go to see the doctor. The universe *U* = {fever  (*x*_1_), headache  (*x*_2_), stomachache  (*x*_3_), cough  (*x*_4_), and chest  pain  (*x*_5_)} is a symptom set and the universe *V* = {viral  fever  (*y*_1_), dysentery  (*y*_2_), typhoid  fever  (*y*_3_), gastritis  (*y*_4_), and pneumonia  (*y*_5_)} is a disease set.  Table 1 is the conditions of five patients and Table 2 is the relation from *U* to *V* determined by medical specialist in advance. Then, the triple (*U*, *V*, *R*) formed a generalized approximation space over two universes.

In the following, we will rank the patients according the method proposed above in order to cure the severe patients earlier.

(1) Weigh Atanassov's intuitionistic fuzzy sets of patients' symptoms. The results are presented in Table 3.

(2) Compute the lower and upper approximations of Atanassov's intuitionistic fuzzy sets in Table 3. Then, Atanassov's intuitionistic fuzzy sets on universe *U* are transformed into Atanassov's intuitionistic fuzzy sets on universe *V*, and Table 4 is the calculation of lower approximations, and Table 5 is the calculation of upper approximations.

(3) Because some diseases are very serious, they must be cured at once, while some diseases are chronic and they can be cured earlier or later. So, the hospital can weigh diseases so that the doctors know the patients' conditions integrally. This weight can be decided by mathematical methods, such as statistical method and differential method. For convenience, we invite experts who have rich experience to score the weighing value. Assume weighing vector of the diseases is *w* = (0.235,0.215,0.185,0.175,0.19).

(4) Now, we can compute the comprehensive attribute value of five patients. According to the project we proposed above, obviously, we have

(50) d(R_U(A~1ω)) =(1−∏i=1n(1−μR_U(A~1ω)(yi))nwi,∏i=1n(νR_U(A~1ω)(yi))nwi) =(0.3392,0.3945).

Similarly,

(51) $$\begin{array}{c} d\left({\underline{R}}_{U}\left({\tilde{A}}_{2\omega }\right)\right)=\left(0.4111,0.3919\right),\\ d\left({\underline{R}}_{U}\left({\tilde{A}}_{3\omega }\right)\right)=\left(0.4656,0.3832\right),\\ d\left({\underline{R}}_{U}\left({\tilde{A}}_{4\omega }\right)\right)=\left(0.3702,0.3776\right),\\ d\left({\underline{R}}_{U}\left({\tilde{A}}_{5\omega }\right)\right)=\left(0.4210,0.4793\right). \end{array}$$

The comprehensive attribute value about upper approximation can be obtained as follows:

(52) d(R¯U(A~1ω))=(1−∏i=1n(1−μR¯U(A~1ω)(yi))nwi) −(∏i=1n(νR¯U(A~1ω)(yi))nwi)=(0.6121,0.1907).

Similarly,

(53) $$\begin{array}{c} d\left({\bar{R}}_{U}\left({\tilde{A}}_{2\omega }\right)\right)=\left(0.6992,0.1519\right),\\ d\left({\bar{R}}_{U}\left({\tilde{A}}_{3\omega }\right)\right)=\left(0.5861,0.2308\right),\\ d\left({\bar{R}}_{U}\left({\tilde{A}}_{4\omega }\right)\right)=\left(0.8069,0\right),\\ d\left({\bar{R}}_{U}\left({\tilde{A}}_{5\omega }\right)\right)=\left(0.6256,0.1883\right). \end{array}$$

(5) We calculate the comprehensive score value in the following:

(54) s(R_U(A~1ω))=(1−∏i=1n(1−μR_U(A~1ω)(yi))nwi) −(∏i=1n(νR_U(A~1ω)(yi))nwi)=−0.0553.

Similarly,

(55) $$\begin{array}{c} s\left({\underline{R}}_{U}\left({\tilde{A}}_{2\omega }\right)\right)=0.0192,\;\;s\left({\underline{R}}_{U}\left({\tilde{A}}_{3\omega }\right)\right)=0.0824,\\ s\left({\underline{R}}_{U}\left({\tilde{A}}_{4\omega }\right)\right)=-0.0074,\;\;s\left({\underline{R}}_{U}\left({\tilde{A}}_{5\omega }\right)\right)=-0.0583. \end{array}$$

According to the lower approximation, that is to say, the first type of projects, the patients' order should be ${\tilde{A}}_{3}$, ${\tilde{A}}_{2}$, ${\tilde{A}}_{4}$, ${\tilde{A}}_{1}$, ${\tilde{A}}_{5}$ because of 0.0824 > 0.0192 > −0.0074 > −0.0553 > −0.0583. This project is very strict with patients, they must know themselves well.

The second type of project is about upper approximation. This project is suitable for the situation in which the patients do not know their condition well. By the similar way, we can calculate the comprehensive score value as follows:

(56) s(R¯U(A~1ω))=(1−∏i=1n(1−μR¯U(A~1ω)(yi))nwi) −(∏i=1n(νR¯U(A~1ω)(yi))nwi)=0.4214.

Similarly,

(57) $$\begin{array}{c} s\left({\bar{R}}_{U}\left({\tilde{A}}_{2\omega }\right)\right)=0.5473,\;\;s\left({\bar{R}}_{U}\left({\tilde{A}}_{3\omega }\right)\right)=0.3553,\\ s\left({\bar{R}}_{U}\left({\tilde{A}}_{4\omega }\right)\right)=0.8069,\;\;s\left({\bar{R}}_{U}\left({\tilde{A}}_{5\omega }\right)\right)=0.4373. \end{array}$$

So the patients' order should be ${\tilde{A}}_{4}$, ${\tilde{A}}_{2}$, ${\tilde{A}}_{5}$, ${\tilde{A}}_{1}$, ${\tilde{A}}_{3}$.

## 6. Conclusions

In this paper, we have introduced rough Atanassov's intuitionistic fuzzy sets model over two different universes in the generalized approximation space (*U*, *V*, *R*). Then, the properties of lower and upper approximation operators are discussed. Meantime, we gave the definitions and properties about rough Atanassov's intuitionistic fuzzy cut sets in the generalized approximation space (*U*, *V*, *R*). Afterwards, we represented how to measure rough Atanassov's intuitionistic fuzzy sets over two different universes. In addition, we also studied an example to illustrate our results. Finally, we designed a new method about how to arrange the patients reasonably so that it can take most of patients into account; this issue is meaningful and useful in our real life.

## Figures and Tables

**Table 1:** The conditions of five patients.

$\langle x_{i},\mu_{{\tilde{A}}_{i}}(x_{i}),\nu_{{\tilde{A}}_{i}}(x_{i})\rangle$	*x*_1_	*x*_2_	*x*_3_	*x*_4_	*x*_5_
${\tilde{A}}_{1}$	(0.3,0.5)	(0.7,0.1)	(0.4,0.2)	(0.8,0.1)	(0.2,0.6)
${\tilde{A}}_{2}$	(0.6,0.2)	(0.5,0.3)	(0.7,0.2)	(0.7,0.1)	(0.3,0.6)
${\tilde{A}}_{3}$	(0.4,0.4)	(0.8,0.1)	(0.5,0.2)	(0.6,0.3)	(0.4,0.5)
${\tilde{A}}_{4}$	(0.3,0.4)	(0.6,0.2)	(0.9,0.0)	(0.8,0.1)	(0.3,0.5)
${\tilde{A}}_{5}$	(0.5,0.3)	(0.3,0.6)	(0.6,0.2)	(0.7,0.2)	(0.6,0.3)

**Table 2:** The relations between symptoms and diseases.

(*U*, *V*, *R*)	*y*_1_	*y*_2_	*y*_3_	*y*_4_	*y*_5_
*x*_1_	1	1	0	0	0
*x*_2_	1	0	1	0	0
*x*_3_	0	0	1	1	0
*x*_4_	1	1	0	0	0
*x*_5_	0	0	0	1	1

**Table 3:** The weighted conditions of five patients.

$\langle x_{i},\mu_{{\tilde{A}}_{i\omega }}(x_{i}),\nu_{{\tilde{A}}_{i\omega }}(x_{i})\rangle$	*x*_1_	*x*_2_	*x*_3_	*x*_4_	*x*_5_
${\tilde{A}}_{1\omega }$	(0.435,0.330)	(0.595,0.178)	(0.444,0.157)	(0.765,0.126)	(0.125,0.736)
${\tilde{A}}_{2\omega }$	(0.770,0.076)	(0.405,0.405)	(0.750,0.157)	(0.662,0.126)	(0.193,0.736)
${\tilde{A}}_{3\omega }$	(0.558,0.231)	(0.701,0.178)	(0.549,0.157)	(0.562,0.338)	(0.264,0.660)
${\tilde{A}}_{4\omega }$	(0.435,0.231)	(0.497,0.299)	(0.929,0)	(0.765,0.126)	(0.193,0.660)
${\tilde{A}}_{5\omega }$	(0.670,0.146)	(0.235,0.682)	(0.651,0.157)	(0.662,0.235)	(0.423,0.486)

**Table 4:** The lower approximations of weighted Atanassov's intuitionistic fuzzy sets.

$\langle y_{i},\mu_{{\underline{R}}_{U}({\tilde{A}}_{i\omega })}(y_{i}),\nu_{{\underline{R}}_{U}({\tilde{A}}_{i\omega })}(y_{i})\rangle$	*y*_1_	*y*_2_	*y*_3_	*y*_4_	*y*_5_
${\underline{R}}_{U}({\tilde{A}}_{1\omega })$	(0.435,0.330)	(0.435,0.330)	(0.444,0.178)	(0.125,0.736)	(0.125,0.736)
${\underline{R}}_{U}({\tilde{A}}_{2\omega })$	(0.405,0.405)	(0.662,0.126)	(0.405,0.405)	(0.193,0.736)	(0.193,0.736)
${\underline{R}}_{U}({\tilde{A}}_{3\omega })$	(0.558,0.338)	(0.558,0.338)	(0.549,0.178)	(0.264,0.660)	(0.264,0.660)
${\underline{R}}_{U}({\tilde{A}}_{4\omega })$	(0.435,0.299)	(0.435,0.231)	(0.497,0.299)	(0.193,0.660)	(0.193,0.660)
${\underline{R}}_{U}({\tilde{A}}_{5\omega })$	(0.235,0.682)	(0.662,0.235)	(0.235,0.682)	(0.423,0.486)	(0.423,0.486)

**Table 5:** The upper approximations of weighted Atanassov's intuitionistic fuzzy sets.

$\langle y_{i},\mu_{{\bar{R}}_{U}({\tilde{A}}_{i\omega })}(y_{i}),\nu_{{\bar{R}}_{U}({\tilde{A}}_{i\omega })}(y_{i})\rangle$	*y*_1_	*y*_2_	*y*_3_	*y*_4_	*y*_5_
${\bar{R}}_{U}({\tilde{A}}_{1\omega })$	(0.765,0.126)	(0.765,0.126)	(0.595,0.157)	(0.444,0.157)	(0.125,0.736)
${\bar{R}}_{U}({\tilde{A}}_{2\omega })$	(0.770,0.076)	(0.770,0.076)	(0.750,0.157)	(0.750,0.157)	(0.193,0.736)
${\bar{R}}_{U}({\tilde{A}}_{3\omega })$	(0.701,0.178)	(0.562,0.231)	(0.701,0.157)	(0.549,0.157)	(0.264,0.660)
${\bar{R}}_{U}({\tilde{A}}_{4\omega })$	(0.765,0.126)	(0.765,0.126)	(0.929,0)	(0.929,0)	(0.193,0.660)
${\bar{R}}_{U}({\tilde{A}}_{5\omega })$	(0.670,0.146)	(0.670,0.146)	(0.651,0.157)	(0.651,0.157)	(0.423,0.486)

## References

[1] Pawlak Z (1982). Rough sets. *International Journal of Computer & Information Sciences*.

[2] Pawlak Z (1991). *Rough Set: Theoretical Aspects of Reasoning about Data*.

[3] Pawlak Z, Sowinski R (1994). Rough set approach to multi-attribute decision analysis. *European Journal of Operational Research*.

[4] Ananthanarayana VS, Murty MN, Subramanian DK (2003). Tree structure for efficient data mining using rough sets. *Pattern Recognition Letters*.

[5] Cheng W, Mo Z, Wang J (2007). Notes on the lower and upper approximations in a fuzzy group and rough ideals in semigroups. *Information Sciences*.

[6] Dempster AP (1967). Upper and lower probabilities induced by a multivalued mapping. *The Annals of Mathematical Statistics*.

[7] Düntsch I, Gediga G (1998). Uncertainty measures of rough set prediction. *Artificial Intelligence*.

[8] Jensen R, Shen Q (2007). Fuzzy-rough sets assisted attribute selection. *IEEE Transactions on Fuzzy Systems*.

[9] Mi J, Zhang W (2004). An axiomatic characterization of a fuzzy generalization of rough sets. *Information Sciences*.

[10] Pawlak Z (2002). Rough sets, decision algorithms and Bayes’ theorem. *European Journal of Operational Research*.

[11] Pawlak Z (2005). Some remarks on conflict analysis. *European Journal of Operational Research*.

[12] Pawlak Z, Skowron A (2007). Rudiments of rough sets. *Information Sciences*.

[13] Zhou L, Wu W (2011). Characterization of rough set approximations in Atanassov intuitionistic fuzzy set theory. *Computers and Mathematics with Applications*.

[14] Zhang Z (2010). An interval-valued rough intuitionistic fuzzy set model. *International Journal of General Systems*.

[15] Xu W, Wang Q, Zhang X (2011). Multi-granulation fuzzy rough sets in a fuzzy tolerance approximation space. *International Journal of Fuzzy Systems*.

[16] Xu WH, Liu YF, Sun WX Uncertainty measure of intuitionistic fuzzy T equivalence information systems.

[17] Shen Y, Wang F (2011). Variable precision rough set model over two universes and its properties. *Soft Computing*.

[18] Yan R, Zheng J, Liu J, Zhai Y (2010). Research on the model of rough set over dual-universes. *Knowledge-Based Systems*.

[19] Sun BZ, Ma WM, Liu Q (2013). An approach to decision making based on intuitionistic fuzzy rough sets over two universes. *Journal of the Operational Research Society*.

[20] Li T, Zhang W (2008). Rough fuzzy approximations on two universes of discourse. *Information Sciences*.

[21] Liu G (2010). Rough set theory based on two universal sets and its applications. *Knowledge-Based Systems*.

[22] Xu WH, Liu YF, Sun WX (2014). Uncertainty measure of Atanassov's intuitionistic fuzzy *T* equivalence information systems. *Journal of Intelligent and Fuzzy Systems*.

[23] Shu L, He XZ Rough set model with double universe of discourse.

[24] Sun B, Ma W (2011). Fuzzy rough set model on two different universes and its application. *Applied Mathematical Modelling*.

[25] Zhang H, Zhang W, Wu W (2009). On characterization of generalized interval-valued fuzzy rough sets on two universes of discourse. *International Journal of Approximate Reasoning*.

[26] Atanassov KT (1986). Intuitionistic fuzzy sets. *Fuzzy Sets and Systems*.

[27] Atanassov K (1999). *Intuitionistic Fuzzy Sets: Theory and Application*.

[28] Dubois D, Prade H (1990). Rough fuzzy sets and fuzzy rough sets. *International Journal of General Systems*.

[29] Xu WH, Sun WX, Liu YF, Zhang WX (2012). Fuzzy rough set models over two universes. *International Journal of Machine Learning and Cybernetics*.

[30] Xu Z (2007). Intuitionistic fuzzy aggregation operators. *IEEE Transactions on Fuzzy Systems*.

